# Comparison of cell response to chromatin and DNA damage

**DOI:** 10.1093/nar/gkad865

**Published:** 2023-10-19

**Authors:** Artyom Luzhin, Priyanka Rajan, Alfiya Safina, Katerina Leonova, Aimee Stablewski, Jianmin Wang, Denisha Robinson, Natalia Isaeva, Omar Kantidze, Katerina Gurova

**Affiliations:** Department of Cellular Genomics, Institute of Gene Biology of the Russian Academy of Sciences, Moscow 119334, Russia; Department of Cell Stress Biology, Roswell Park Comprehensive Cancer Center, Elm and Carlton Sts, Buffalo, NY 14263, USA; Department of Cell Stress Biology, Roswell Park Comprehensive Cancer Center, Elm and Carlton Sts, Buffalo, NY 14263, USA; Department of Cell Stress Biology, Roswell Park Comprehensive Cancer Center, Elm and Carlton Sts, Buffalo, NY 14263, USA; Gene Targeting and Transgenic Shared Resource, Roswell Park Comprehensive Cancer Center, Elm and Carlton Sts, Buffalo, NY 14263, USA; Department of Bioinformatics, Roswell Park Comprehensive Cancer Center, Elm and Carlton Sts, Buffalo, NY 14263, USA; Department of Cell Stress Biology, Roswell Park Comprehensive Cancer Center, Elm and Carlton Sts, Buffalo, NY 14263, USA; Department of Otolaryngology/Head and Neck Surgery; Lineberger Comprehensive Cancer Center, University of North Carolina, Chapel Hill, NC 27599, USA; Quantori LLC, Cambridge, MA 02142, USA; Department of Cell Stress Biology, Roswell Park Comprehensive Cancer Center, Elm and Carlton Sts, Buffalo, NY 14263, USA

## Abstract

DNA-targeting drugs are widely used for anti-cancer treatment. Many of these drugs cause different types of DNA damage, i.e. alterations in the chemical structure of DNA molecule. However, molecules binding to DNA may also interfere with DNA packing into chromatin. Interestingly, some molecules do not cause any changes in DNA chemical structure but interfere with DNA binding to histones and nucleosome wrapping. This results in histone loss from chromatin and destabilization of nucleosomes, a phenomenon that we call chromatin damage. Although the cellular response to DNA damage is well-studied, the consequences of chromatin damage are not. Moreover, many drugs used to study DNA damage also cause chromatin damage, therefore there is no clarity on which effects are caused by DNA or chromatin damage. In this study, we aimed to clarify this issue. We treated normal and tumor cells with bleomycin, nuclease mimicking drug which cut predominantly nucleosome-free DNA and therefore causes DNA damage in the form of DNA breaks, and CBL0137, which causes chromatin damage without direct DNA damage. We describe similarities and differences between the consequences of DNA and chromatin damage. Both agents were more toxic for tumor than normal cells, but while DNA damage causes senescence in both normal and tumor cells, chromatin damage does not. Both agents activated p53, but chromatin damage leads to the accumulation of higher levels of unmodified p53, which transcriptional activity was similar to or lower than that of p53 activated by DNA damage. Most importantly, we found that while transcriptional changes caused by DNA damage are limited by p53-dependent activation of a small number of p53 targets, chromatin damage activated many folds more genes in p53 independent manner.

## Graphical Abstract

**Graphical Abstract:**
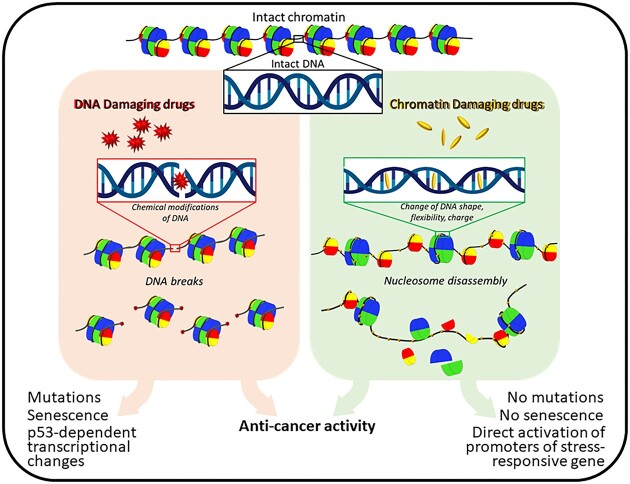

## Introduction

DNA-binding small molecules have been used in medicine and biology for a long time. They showed multiple clinical and biological effects, including anti-cancer, anti-infective, and anti-inflammatory activities. Most of their effects are attributed to their ability to cause DNA damage (DnaD), i.e. alter DNA chemical structure. In addition, the effects of many of these molecules on the structure and function of chromatin in cells and cell-free conditions were observed long ago (1,2); however, chromatin-related effects were not investigated in depth because their biological significance and impact on the anti-cancer activity were unknown. Recently, the chromatin-related effects of DNA binding drugs have been getting more attention, with a focus on separating the cellular consequences of DnaD from the chromatin alterations caused by these molecules. Two pivotal studies published in 2013 demonstrated that anti-cancer agents from anthracycline group cause histone eviction from chromatin and that this effect does not correlate with DNA-damaging activity of these molecules (3,4). It was proposed that histone eviction may result from their effects on DNA topology (4) and may have implications for the mechanisms underlying cell killing during cancer chemotherapy (3,4).

Our group discovered the curaxins, candidate anti-cancer agents (5), and defined their mechanism of action (5). Curaxins are chemically different from anthracyclines (i.e. carbazole vs. anthraquinone derivatives); however, there are similarities in their DNA binding. Both types of compounds intercalate between DNA base pairs and have their side chains in DNA grooves. Curaxins clinical lead, CBL0137, do not damage DNA in mammalian cells, as judged by the absense of DNA breaks in treated cells (6,7) and absence of activation of DNA-damage sensitive kinase (5). Additional argument confirming insignificance of DnaD from curaxins treatment is that life-long exposure of cancer-prone mice to CBL0137 did not increase (6) or even reduced incidence of cancer in mice (8,9). At the same time curaxins efficiently evict histones from chromatin, an effect we named ‘chromatin damage’ (ChrD) (10,11). Similar to anthracyclines, curaxins have demonstrated anti-tumor activity in multiple preclinical models (i.e. have higher toxicity to tumor cells than normal cells), suggesting that tumor cells are more sensitive to ChrD than normal cells (6–7,9,12–15). The reason behind this phenomenon is unclear.

Curaxin binding to DNA causes the lengthening of the double helix, reduces DNA flexibility and its negative charge (curaxins have a small positive charge), and competes for minor groove binding with histones. However, whether all these effects or just some are important for ChrD and what structural features of these small molecules define the strength of their effect on chromatin are currently unknown. We previously characterized the effect of several known anti-cancer agents differing in their binding to DNA in their ability to cause DnaD, ChrD and compromise tumor cell viability to understand why some compounds cause ChrD and others cause DnaD or both, ChrD and DnaD (11). We found that only compounds binding DNA directly cause ChrD, whereas compounds chemically modifying DNA without direct, stable DNA binding (e.g. bleomycin causes DNA breaks through nuclease-like activity (16–18)), or compounds incorporated into DNA as modified nucleotides, or compounds binding and inhibiting DNA enzymes that use DNA as a substrate (e.g. topoisomerase inhibitor etoposide does not bind DNA per se) do not cause direct ChrD. Moreover, we observed that ChrD activity of small molecules had a higher impact on cytotoxicity than DnaD activity (11). In contrast to ChrD, the cellular stress response to DnaD is well-studied; however, in most studies molecules used to cause DnaD were also causing ChrD, but this effect was not considered. This suggests that some effects attributed to DnaD may, in fact, be caused by ChrD.

A major long-term side effect of anthracycline chemotherapy is cardiotoxicity (17). Recently, Neefjes group (18) demonstrated that cardiotoxicity is caused by compounds that induce strong DnaD (e.g. doxorubicin), whereas similar compounds with very weak DnaD activity (e.g. aclarubicin) have similar anti-cancer effects without cardiotoxicity, suggesting that although cardiotoxicity is a consequence of DnaD, its anti-cancer activity is not (18). Because of these data, it is even more important to separate the cellular consequences of DnaD and ChrD to be able to design and use drugs with maximal anti-cancer activity and minimal long-term toxicity. Here, we used agents that, in the short-term, predominantly cause DnaD (i.e. DNA breaks) or ChrD (i.e. histone eviction from chromatin without DNA breaks) to distinguish between the cellular effects caused by ChrD and those resulting from DnaD. As inducer of ChrD we used curaxins CBL0137. As DnaD inducers we selected glycopeptide antibiotics from Streptomyces verticillus belonging to the bleomycin family. These compounds cause double-strand (ds) DNA breaks (rev. in (19)). The crystal structure of bleomycin with DNA shows that it binds DNA and acts like a nuclease. It has a metal binding domain and causes site-selective cleavage of duplex DNA at 5′-GT/C sites after oxygenation (16,20–21). Importantly, bleomycin does not digest nucleosomal DNA but attacks only nucleosome-free DNA (22).

## Materials and methods

### Reagents

CBL0137 was provided by Incuron, Inc. and dissolved in dimethyl sulfoxide at 20 mM. Bleomycin solution for injection was provided by Roswell Park Pharmacy (leftover from clinical use) as 30 mg/ml solution. In most experiments we used Zeocin (trade name of phleomycin D1, antibiotic structurally similar to bleomycin) after confirming activity identical to bleomycin. It was purchased from Thermo Fisher Scientific (Grand Island, NY) as 50 mg/ml solution. Resazurin sodium salt was purchased from Thermo Fisher Scientific. Resazurin was dissolved in Dulbecco's phosphate-buffered saline (DPBS, pH 7.4) at 0.15 mg/ml (10× solution). ATP-Glo substrate was prepared in house by as 0.0005 mg/ml recombinant Firefly luciferase (Cayman Chemicals, Ann Arbor, MI), 0.25 mM d-luciferin (Sigma Aldrich, St. Louis, MO) and 0.25 mM ATP (Sigma Aldrich, St. Louis, MO) in an assay buffer (50 mM Tris base (pH 7.8), 25 mM NaCl, 1 mM MgCl_2_, 0.1 mM EDTA, 20 mM DTT, 0.25% Triton X100, 0.05% Antifoam 204). Bright-Glo-Luciferase substrate was purchased from Promega Inc. (Fitchburg, WI) or prepared in house from luciferin (GoldBio, Olivette, MO) and ATP (Sigma Aldrich, St. Louis, MO) as described (23). Micrococcal Nuclease was purchased from New England BioLabs (Ipswich, MA). Other chemicals were purchased from Sigma-Aldrich (St. Louis, MO).

### Cells

HT1080, Be2C, MCF7, HCT8 and HT29 cell lines are from the American Type Culture Collection. HT1080 and MCF7 cells were authenticated using short tandem repeat analysis (100% match). Primary human neonatal dermal fibroblasts (NDFs) were obtained from AllCells, LLC (Alameda, CA), as a pool of three separate donors. HT1080 cells expressing H2B-mCherry and H1-mCherry were already described (5,24). HeLa-TI cells expressing epigenetically silenced GFP under CMV promoter were described in (25,26).

HT1080 cells were maintained in Dulbecco's modified Eagle's medium (DMEM) supplemented with 5% (v/v) FBS and 100 units/ml penicillin. Other cells were maintained in DMEM supplemented with 10% (v/v) FBS, 100 units/ml penicillin, 100 μg/ml streptomycin and 2 mM l-glutamine. Cells were cultured in a tissue culture incubator at 37°C and 5% CO_2_.

### Plasmids

The p53-Luc lentiviral plasmid and GSE56 LXSN vector were previously described (27,28). Human high-mobility group box 1 MISSION® shRNA Bacterial Glycerol Stock was purchased from Sigma-Aldrich as five independent clones (SHGLYC-TRCN00000189*30 – 34*). pH2B_mCherry_IRES_neo3 plasmid was a gift from Daniel Gerlich (Addgene plasmid # 21044). HMGB1 lentiviral expressed vector # EX-B0072-LV105 was purchased from GeneCopeoiea (Rockville, MD) Lentivirus production and transduction were done as described (27).Transfection was performed using Lipofectamine™ 3000 (Invitrogen, Thermo Fisher Scientific (Grand Island, NY)) according to manufacturer protocol.

### Deletion of p53 in cells

Four single-guide RNA (sgRNA) sequences were used to target intron 1, exon 3, exon 4 and intron 9 of human p53 to knock out the gene in HT1080 and NDF cells, as previously described (29,30): ACTTCCTGAAAACAACGTTCTGG (e3), GAGCGCTGCTCAGATAGCGATGG (e4), TCTGCAGGCCCAGGTGACCCagg (i1), and GAAACTTTCCA CTTGATAAGagg (i9). Crispr RNA (crRNA) and tracer RNA (trRNA) were purchased from IDT DNA Technologies (Coralville, IA) and resuspended to 160 μM each. crRNA and trRNA (1:1) were complexed using touchdown polymerase chain reaction (PCR) (IDT DNA Technologies), and Cas9 3NLS protein (IDT DNA Technologies) was added to make functional ribonucleoprotein (RNP). RNP was added to 3 × 10^6^ cells suspended in Opti-MEM medium (Thermo Fisher Scientific). The cell mixture was electroporated using a NEPA21 electroporator (Bulldog Bio, Portsmouth, NH) and then transferred into 6-well plates containing complete medium without antibiotics (2 ml). Three electroporation regimens differing in voltage and time of stroke were used with similar results.

After recovery from electroporation, the cells were expanded and frozen. Cells from each electroporation regimen were plated in two plates. One plate was left untreated, and the second plate was treated with 1 μM CBL0137 for 24 h to induce p53. Non-electroporated cells were used as control cells with wild type p53. The next day, cells were stained for p53, and the number of p53-positive cells was assessed by flow cytometry (Supplementary Figure S3). For further experiment we used NDF cell population with ∼60% p53 positive cells (#3 on Supplementary Figure S3). Transfected HT1080 cell populations contained less than 20% of p53 positive cells (Supplementary Figure S3), therefore we added original p53 wild type cells to one of the populations to have 1:1 ratio of p53 positive and negative cells.

In parallel, cells were plated in 96-well plates at 1 cell/well. After colony formation (7–10 days), wells were inspected using microscopy and wells with no colonies or more than one colony were excluded. Cells from other wells were trypsinized and replated into three new 96 well plates. One plate was used as a reference plate and two other plates were either left untreated or treated with 1 μM CBL0137 for 24 h. These two plates were stained for p53 after that, and the p53-positive and p53-negative clones were identified using fluorescence microscopy. All p53-negative clones were pooled. Loss of p53 was confirmed by western blotting under basal and CBL0137-treated conditions.

### Live cell imaging

HT1080 cells expressing H1-mCherry or H2B-mCherry were plated in CultureWell Chambered Coverglass (Grace BioLabs, Bend, OR). For live cell fluorescent image acquisition, cells were treated with drugs and images were obtained at different time points with a Zeiss Axio Observer A1 inverted microscope with N-Achroplan 100×/1.25 oil lens, Zeiss MRC5 camera, and AxioVision Rel.4.8 software. Images of nuclei were slightly re-scaled (no > 30% of originial size) in order to be of approximately the same. Figure panels with re-scaled images are shown without scale bars. Contrast and brightness were adjusted to compensate for the different levels of ectopic histone expression between individual cells.

### Luciferase reporter assay

Cells were plated in 96- or 384-well plates at 5000 or 1500 cells per well in 100 or 40 μl medium. The next day, the same amount of medium with drugs was added to the cells on 96 well plates manually or to 384 well plates using D300e Digital Dispenser (Tecan HP). Cells were incubated with drugs for 24 h in 96-well plates luciferase activity was measured using a BrightGlo kit from Promega (Madison, WI) and in 384 wells using home-made luciferase substrate.

### Viability assay

Cells were plated in 96-well plates at 1000 cells per well in 100 μl medium. The next day, 100 μl medium with drugs was added to the cells for 72 h. The effects of treatment were determined either by adding 20 μl 10× resazurin solution for 4–8 h and reading fluorescence (560/590 nm) or by adding ATP-Glo solution and reading luminescence using a multi-plate reader. Alternatively, cells were plated in 384-well plates. The next day, the cells were treated with different drug concentrations in triplicate. Cell numbers were counted every 12 h using the Cytation 5 Cell Imaging Multimode Reader with Gen5 ImagePrime software (Biotec, Agilent Technologies, Santa Clara, CA).

### Induction of senescence

Cells were plated at 5 × 10^5^ per 10-cm plate and next day treated with CBL0137 or bleomycin for 24 h. The compounds were washed off after treatment, and the cells were cultured for 10 days with medium changes every 72 h. After 10 days, cells were trypsinized and plated for colony formation assay, acidic beta-galactosidase staining or measurement of IL-6 and IL-8 in culture medium.

### Colony formation assay

10 days after treatment with CBL0137 or bleomycin, the cells were trypsinized, seeded at 100 cells per 10-cm plate, and allowed to grow for approximately 2 weeks. Cells were washed with PBS, and stained with 1% methylene blue in methanol. The experiment was performed in triplicate for each condition, and colonies of ≥50 cells were counted by light microscopy at 10× magnification.

### Senescence-associated β-galactosidase staining

10 days after treatment with CBL0137 or bleomycin, the cells were trypsinized and seeded at 12×10^3^ cells per well in 12-well plates. After 24 h, the cells were fixed, and stained for acidic β-galactosidase activity as previously described (31). Briefly, cells were washed twice in PBS, fixed for 5 min at room temperature with 2% (v/v) paraformaldehyde and 0.2% (v/v) glutaraldehyde, washed with PBS and then incubated with freshly prepared SA-β-Gal staining solution (1mg/ml X-Gal, Thermo Fisher Scientific) dissolved in *N*,*N*-dimethylformamide (Sigma-Aldrich), 100 mM citric acid/sodium phosphate buffer (pH 6.0) containing 2 mM MgCl_2_, 150 mM NaCl, 5 mM K_3_Fe(CN)_6_ and 5 mM K_4_Fe(CN)_6_. Staining was performed at 37°C in a non-CO_2_ incubator for up to 18 h until the X-Gal product was visible. The reaction was stopped by washing the cells with PBS.

### Measurement of IL-6 and IL-8 in culture medium

IL-6 and IL-8 were measured in culture medium using enzyme-linked immunosorbent assays (ELISA). 10 days after treatment with CBL0137 or bleomycin, the cells were trypsinized and seeded at 12 × 10^3^ cells per well in 12-well plates in 1 ml complete medium. After 72 h, the conditioned medium was collected and frozen at -80°C. The concentrations of IL-6 and IL-8 were measured in triplicate with the DuoSet ELISA Development System (R&D Systems, Minneapolis, MN) according to the manufacturer's instructions. Cytokine levels were normalized to the cell number for each condition.

### Comet assay (single-cell gel electrophoresis)

Glass slides were coated with 1% low melting point agarose (LMPA, Type 1A low electroendosmosis agarose (Sigma-Aldrich, St. Louis, MO, cat # A0169) in MilliQ water (Sigma-Aldrich, St. Louis, MO). Once solidified, two wells were created using the top of a 1-ml pipette tip. The slides were left at room temperature overnight in a sealed box to avoid evaporation. Treated and untreated cells were trypsinized and then resuspended in 37°C 1% LMPA at a concentration of 1.1 ×10^6^ cells/ml (50 000 cells per 90 μl per sample). The cell-agarose suspension (90 μl) was added to each well of the slides, which were chilled on ice. After a gel formed, a layer of 0.5% LMPA (100 μl) was added to the wells and allowed to solidify.

The slides were placed in freshly made lysis buffer (2.5 M NaCl, 100 mM EDTA, 10 mM tris base, pH 10, 1% Triton X-100) for 2.5 h at 4°C, protected from light. The slides were then placed in an alkaline solution (12 g/l NaOH, 500 mM EDTA) at 4°C for 30 min, protected from light. After removing the alkaline solution, the slides were immersed twice in 1 × TBE electrophoresis buffer for 5 min each. Electrophoresis was performed at 24 V (0.74 V/cm) for 30 min. After electrophoresis, the slides were washed thrice in prechilled water for 2 min each. The slides were dehydrated with ice-cold 70% ethanol for 5 min, allowed to air dry, and then stained with Vista Green (Cell Biolabs, San Diego, CA, cat # 235005) diluted 1:10 000 in TE buffer. Comets were scored using the OpenComet plugin from ImageJ. The percentage of tail DNA across various samples was determined. The fraction of cells with comets was calculated to determine the prevalence of DNA damage across the samples.

Pulse field gel electrophoresis was done as previously described (7).

### Immunoblotting

Soluble protein extracts were prepared by incubation of cells in 1 × Cell Culture Lysis Reagent (Promega, Madison, WI) with Complete Protease Inhibitor Cocktail (Roche) on ice for 20 min. Samples were centrifuged at 4°C at 13 000 rpm for 20 min, and the supernatant was collected. Proteins from pellet were solubilized by resuspension of the remaining pellets in 1 × Cell Culture Lysis Reagent (Promega, Madison, WI) and sonicated three times for 30 s on ice using Bioruptor UCD-200 (Diagenode). For measuring of histones in pellet fraction (chromatin) before sonication pellets were washed with 400 mM NaCl. For total cell extracts cell lysates were not centrifuged but instead sonicated the same way as pellet fraction. Western blotting was performed as described (11) using the following antibodies

**Table:** 

Antibody	Company	Catalog #	Dilution
P53 DO-1	Santa Cruz Biotechnology	sc-126	1:1000 (WB), 1:200 (IF)
P21 F-5	Santa Cruz Biotechnology	sc-6246	1:200 (WB)
P21 12D1	Cell Signaling Technologies	2947S	1:2000 (WB)
MDM2 SMP14	Santa Cruz Biotechnology	sc-965	1:200 (WB)
SSRP1	BioLegend	609702	1:4000 (WB)
SPT16	BioLegend	607002	1:1000 (WB)
γH2AX	Cell Signaling Technologies	9718S	1:1000 (WB)
p-p53 (serine 15)	Cell Signaling Technologies	9286P	1:200 (IF)
p-CHK1 (serine 317)	Cell Signaling Technologies	2349P	1:200 (IF)
CHK1	Cell Signaling Technologies	2360S	1:200 (IF)
p-BRCA1 (serine 1524)	Cell Signaling Technologies	9009	1:200 (IF)
RPA1	Cell Signaling Technologies	2267	1:200 (IF)
XRCC1	Abcam	ab1838	1:200 (IF)
HMGB1	Cell Signaling Technologies	3935	1:1000 (WB)
H3	Sigma- Aldrich	06–755	1:3000 (WB)
H1	Cell Signaling	41328	1:1000 (WB)
Beta-Actin	Sigma-Aldrich	A3854	1:30 000 (WB)
GAPDH	Invitrogen	MA5-15738	1:10 000 (WB)
Anti-mouse HRP	Cell Signaling Technologies	sc-516102	1:5000 (WB)
Anti-rabbit HRP	Santa Cruz Biotechnology	sc-2357	1:4000 (WB)
Anti-mouse Alexa-Fluor488	Invitrogen	A21202	1:500 (IF)
Anti-rabbit Alexa Fluor594	Invitrogen	A-11012	1:500 (IF)

### p53 staining for flow cytometry

Cell suspensions (1×10^5^ cells in 300 μl 3% BSA in PBS) or adherent cells on a p60 plate were fixed in 1 ml 4% paraformaldehyde (PFA) for 10 min at room temperature. After fixing, the cells were washed thrice with PBS, blocked with 3% BSA in PBS containing 0.1% Triton X-100 for 1 h, and stained with primary and secondary antibodies for 1 h each. The cells were washed thrice with 0.1% Triton X-100 in PBS after each antibody incubation. Resuspended cells were analyzed by flow cytometry, and the adherent cells were assessed using a Zeiss AxioVision A1 fluorescent microscope.

### 5-Ethynyl-2′-deoxyuridine (EdU) incorporation assay

Cells were treated with 1 μM CBL0137 or 500 μg/ml bleomycin for 24 h and then incubated with 10 μM of EdU for 2 h. After treatment, the cells were fixed with 4% PFA for 15 min. EdU incorporation was detected with the Click-IT Plus EdU Flow Cytometry Assay (Invitrogen, cat#10632) using the manufacturer's staining protocol.

### 5-Ethynyl uridine (EU) incorporation assay

Cells were treated with CBL0137 (0, 0.25, 0.5 and 1 μM) for 3 hr, with EU (0.2 mM) added for the last hour. After treatment, the cells were fixed with 4% PFA for 15 min, and EU incorporation was detected using reagents from the Click-IT Plus EdU Flow Cytometry Assay following the EdU protocol.

### MNase digestion

Cells were treated with 1 μM CBL0137 or 500 μg/ml bleomycin for 24 h and then nuclei of cells were isolated and incubated with micrococcal nuclease followed by DNA isolation and agarose gel electrophoresis as described (5).

### Nascent RNA sequencing

HT1080 cells were treated with 400 μg/ml bleomycin or 0.5 μM CBL0137 in triplicate. After 24 h, cells were incubated with 1 mM EU for 20 min. RNA was isolated using the Monarch Total RNA Miniprep Kit (New England BioLabs, cat #T2010) following the manufacturer's protocol. Nascent RNA was labeled with biotinylated azide and captured on magnetic beads using the Click-iT Nascent RNA Capture Kit (Molecular Probes, cat # C10365). cDNA was synthesized from RNA bound to the beads using the SuperScript VILO cDNA Synthesis Kit (ThermoFisher Scientific, cat #11754-050). cDNA was sequenced with the Illumina NextSeq 500/550 at the Roswell Park Comprehensive Cancer Center Genomics Shared Resource using Illumina NextSeq 500/550.

### Cell treatment for single-cell RNA sequencing

Populations of NDFs or HT1080 cells expanded after electroporation with approximately 50% of 53-positive and p53-negative cells were treated with 1 μM CBL0137 or 500 μg/ml bleomycin for 24 h and then labeled with hashtag antibody oligonucleotide conjugate (HOT) from Biolegend (San Diego, CA) as follows: untreated cells, TotalSeq™-B0258 anti-human Hashtag 8 antibody; CBL0137-treated cells, TotalSeq™-B0259 anti-human Hashtag 9 antibody; bleomycin-treated cells, TotalSeq™-B0260 anti-human Hashtag 10 antibody. The different treatment groups for a given cell type were processed and sequenced together as one sample. Antibody binding and stability at the cell surface over several h were tested by flow cytometry prior to the experiment.

### 10× Genomics

Single-cell libraries were generated using the 10× Genomics platform. Cell suspensions were assessed by trypan blue using the Countess FL automated cell counter (Thermo Fisher Scientific) to determine the concentration, viability, and absence of clumps and debris that could interfere with single-cell capture. Cells were loaded into the Chromium Controller (10× Genomics), where they were partitioned into nanoliter-scale gel beads-in-emulsion with a single barcode per cell. Reverse transcription was performed, and the resulting cDNA was amplified. The amplified full-length cDNA was used to generate gene expression libraries by enzymatic fragmentation, end-repair, a-tailing, adapter ligation, and PCR to add Illumina-compatible sequencing adapters. The resulting libraries were evaluated with D1000 ScreenTape using a TapeStation 4200 (Agilent Technologies) and quantitated using the Kapa Biosystems qPCR quantitation kit for Illumina. The libraries were pooled, denatured, and diluted to 300 pM with 1% PhiX control library added. The resulting pool was loaded into the appropriate NovaSeq Reagent cartridge and sequenced on a NovaSeq6000 following the manufacturer's recommended protocol (Illumina Inc.).

### MNase-sequencing

Micrococcal nuclease (MNase) was purchased from Worthington Biochemical Corp (Lakewood, NJ, Cat# LS004797). MNase digestion was done as described (32) followed by the analysis of fragment length by BioAnalyzer (Agilent Technologies, Inc., Santa Clara, CA). Digestion conditions resulting in predominantly monomucleosome fragments of 150–200 bp were used for DNA isolation. The sequencing libraries were prepared with the HyperPrep Kit (KAPA BIOSYSTEMS), from 1ug DNA. Following manufacturer's instructions, the first step repairs the ends of the DNA fragments and a single ‘A’ nucleotide is then added to the 3′ ends of the blunt fragments. Indexing adapters, containing a single ‘T’ nucleotide on the 3′ end of the adapter, are ligated to the ends of the dsDNA, preparing them for hybridization onto a flow cell. Adapter ligated libraries are amplified by 3 cycles of PCR, purified using AMPureXP Beads (Beckman Coulter), and validated for appropriate size on a 4200 TapeStation D1000 Screentape (Agilent Technologies, Inc.). The DNA libraries are quantitated using KAPA Biosystems qPCR kit, and are pooled together in an equimolar fashion, following experimental design criteria. Each pool is denatured and diluted to 300 pM with 1% PhiX control library added. The resulting pool is then loaded into the appropriate NovaSeq Reagent cartridge for 50 cycle paired-end reads and sequenced on a NovaSeq6000 following the manufacturer's recommended protocol (Illumina Inc.).

### Bioinformatics analyses

Nascent RNA-seq paired-end raw sequencing reads passed quality filter from Illumina RTA were first pre-processed by using FastQC (v0.11.5) (33) for sequencing base quality control. Reads were mapped to the Human Reference Genome (NCBI Build 37, GRCh37 (hg19)) using BWA (v0.7.15) (34). After marking PCR duplicates using Picard tool (v2.20.5, Broad Institute, Cambridge, MA) a second pass QC was done using alignment output with RSeQC (v2.6.3) (35) to examine abundances of genomic features and gene-body coverage. Gene expression is quantified according to hg19 RefSeq annotation using featureCounts from Subread aligner (v1.6.0) (36) with –fracOverlap 1 option. Differential expression analyses were performed using DESeq2 (v1.18.1) (37), a variance-analysis package developed to infer the statically significant difference in RNA-seq data. Downstream and visualization plots were done using regularized-log_2_ transformation implemented by DESeq2. For the scRNA-seq Chromium 10× Genomics libraries, raw sequencing data were processed using Cellranger software (http://software.10xgenomics.com/single-cell/overview/welcome) with GRCh37 (hg19) reference genome and GENCODE annotation. cloupe files generated by Cellranger was used with desktop Loupe Browser (10 × Genomics) for cell filtering, analyses and visualization. First, data were filtered using the following thresholds: Unique Molecular Identifier (UMI) per barcode (cell) 30000–130 000, features (genes) per barcode (cell)—6000–8000, maximal mitochondrial counts – 7.5%. Then, cells were demultiplexed with hashtag oligos (HTOs) and assigned to the corresponding sample using threshold of Log2 > 8 for of each antibody barcode count. Then, cells were assigned as p53 positive if *TP53* counts > 0 (linear) and *MDM2* counts log_2_ > 1 and as p53 negative if *TP53* counts = 0 (linear) and *MDM2* counts log_2_ ≤ 1. Gene Set Enrichment Analysis was done using MSigDB online resource (Broad Institute, Cambridge, MA).

Raw GRO-seq reads from GSM1055806 were mapped to the Human Reference Genome (NCBI Build 38 (hg38)) using STAR (38). The resulting sequence alignment map (SAM) files were filtered using mapping quality (MAPQ > 10), sorted, and converted to the BAM format using samtools 1.10 (39). Nascent transcription profiles and metagene plots were generated using deepTools 2.0 (40). The pausing index was calculated as the ratio of the density of the reads near the transcription start site (TSS) (+50 bp downstream) to the density of the reads in the whole gene.

Raw MNase-seq reads were mapped to the human genome hg38 using Bowtie2 (41) with parameter –very-sensitive. PCR duplicates in the alignments were filtered using the rmdup command from samtools 1.10. Sam files were sorted and converted to bam files using samtools 1.10. Bigwig files and metagene plots were generated using deepTools 2.0.

ChIP-seq and DNAse I sensitivity datasets for NDFs (ENCFF302JEV, ENCFF241ZSS, ENCFF087PPB, ENCFF715BVU, ENCFF969ORH, ENCFF471BTF, ENCFF429ZOO, ENCFF751GHM, ENCFF684QGK and ENCFF087BQO) were downloaded from the ENCODE Project.

Statistical analyses were performed using GraphPad Prism software. The methods used for the analyses if individual experiments are indicated in the figure legends.

## Results

### Confirmation of the specific activity of the selected inducers of DNA damage and chromatin damage

Nuclease mimetic, bleomycin but not CBL0137, within 24 h of exposure caused DNA damage (DnaD), detected in the comet assay (Supplementary Figure S1A) and by staining for markers of DNA breaks, such as phosphorylated histone H2AX (γH2AX), BRCA1 at serine 1524, CHK1 at serine 317 and p53 at serine 15 (Supplementary Figure S1C, D, F, G). In contrast, CBL0137, as well as another curaxin CBL0100, did not show DnaD activity in any of these assays, as well as in additional assays including direct measurement of DNA breaks by pulse field gel electrophoresis (Supplementary Figure S1B) and replication dependent DNA damage (staining for XRCC1 and RPA1, Supplementary Figure S1F). At the same time CBL0137 but not bleomycin, caused chromatin damage (ChrD) manifested as time a dose dependent redistribution of mCherry tagged histones H1 or H2B in nuclei of HT1080 (Supplementary Figures S2 and S3), eviction of endogenous histones from chromatin (Supplementary Figure S4A), chromatin trapping (c-trapping) of histone chaperone FACT: FACT subunits, SSRP1 and SPT16 bind unwrapped nucleosomes, and therefore, relocate from the nucleoplasm to the chromatin after CBL0137 treatment (Supplementary Figure S4C) (5). Absence of histone eviction and c-trapping in bleomycin treated cells is shown in Supplementary Figures S4B and D. CBL0137 but not bleomycin also causes loss of nucleosome protected bands of DNA upon incubated of nuclei of treated cells with micrococcal nuclease (MNase, Supplementary Figure S4E, F). Importantly, functional consequences of curaxin treatment, which we observed before (24), was re-expression of epigenetically silenced GFP. This effect was significantly weaker upon bleomycin treatment (Supplementary Figure S4G, H).

We also observed increased toxicity of CBL0137 but not of bleomycin to cells overexpressing HMGB1 protein (Supplementary Figure S5). HMGB1 binds DNA in a nonspecific manner via its two HMGB‐box domains. This allows it to bend and contort DNA from nucleosome at entry and exit sites, thus making chromatin more open (42,43)

Thus, we used bleomycin and CBL0137 for the comparison of cellular consequences of DnaD and ChrD.

### Contrary to DNA damage, chromatin damage does not induce senescence

**Figure 1.**
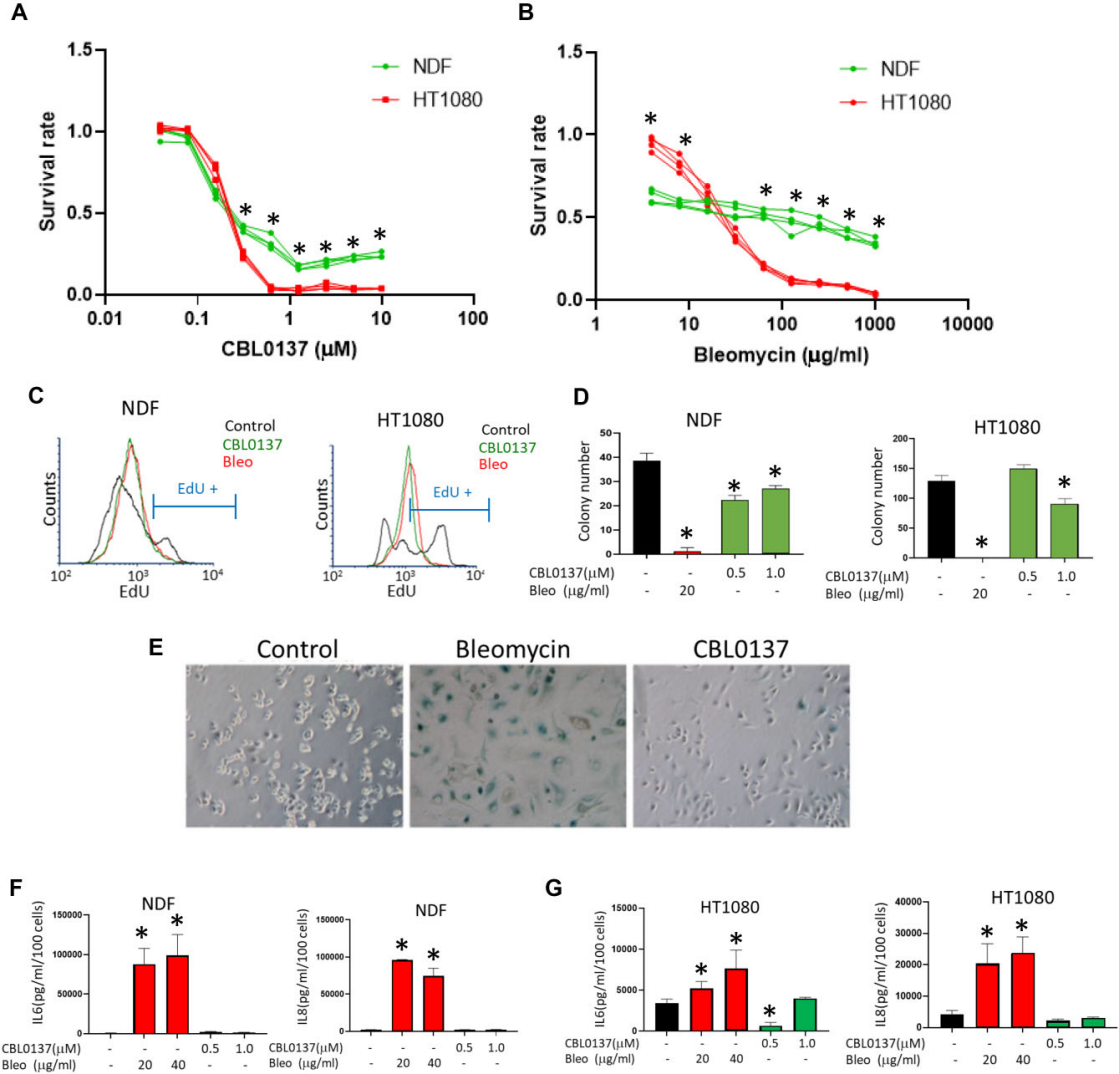
Comparison of the cellular effects of DnaD and ChrD. (A, B) Cytotoxicity of CBL0137 and bleomycin in NDFs (**A**) and HT1080 cells (**B**) after 72 h assessed by resazurin staining. The lines represent four replicates of one representative experiment normalized to untreated cells. *P < 0.05 by the Student's *t*-test, NDFs versus HT1080 cells. (**C**) EdU incorporation assay comparing DNA replication in NDFs and HT1080 cells treated with 500 μg/ml bleomycin (Bleo) or 1 μM CBL0137 for 24 h. EdU was added 2 hr before the cells were fixed and stained. Histograms show the distribution of EdU-positive cells in the different samples. The marker shows the area of EdU-positive cells in untreated samples. (D–G) Assessment of the senescence phenotype in cells treated with bleomycin or CBL0137 for 24 h followed by 10 days in drug-free medium. After 10 days, the cells were split and assessed for the ability to proliferate (colony formation—**D**), acidic beta-galactosidase staining (**E**), and secretion of SASP factors, IL6 (**F**) or IL8 (**G**). (D, F, G) Data are presented as the mean ± SD (*n* = 3). *P < 0.05 by the Student's *t*-test versus untreated cells.

First, we compared time and dose dependent effects of DnaD and ChrD on the viability of two human mesenchymal cell types, normal dermal fibroblasts (NDF) and human fibrosarcoma cell line HT1080. These cells have diploid genome and wild type p53. Both ChrD and DnaD decreased the viability of tumor cells more than normal cells (Figure 1A, B), although this relationship was reversed for bleomycin at lower concentrations (Figure 1B). Interestingly, the transition from no toxicity to full toxicity was much steeper for CBL0137 (occurring between 0.3 and 1 μM) than for bleomycin (toxicity gradually increased through three orders of magnitude from 1 to 1000 μg/ml) (Figure 1A, B and Supplementary Figure S6A, B). DnaD and ChrD suppressed DNA replication to a similar extent (Figure 1C).

DnaD and ChrD differed in their ability to cause senescence. Bleomycin is the most frequently used chemical to induce senescence *in vitro* and *in vivo* (44). Consistent with these findings, we observed the emergence of senescent markers in cells treated with bleomycin dose as low as 20 μg/ml. In contrast, low CBL0137 doses did not affect cell growth, whereas higher doses caused cell death within 48 hr. At a narrow range of concentrations (0.5–1 μM) and limited time of incubation (up to 24 h), CBL0137 caused growth arrest; however, these cells were able to proliferate and form colonies from single cells after the compound was washed out (Figure 1D). Cells arrested by CBL0137 did not develop markers typical for senescent cells, such as positive staining for acidic beta-galactosidase (Figure 1E) or senescence-associated secretory phenotype (Figure 1F, G). Thus, major difference between DnaD and ChrD in their cytotoxic effects was inability of ChrD to induce senescence.

### Different dynamics of p53 activation by DNA and chromatin damage

**Figure 2.**
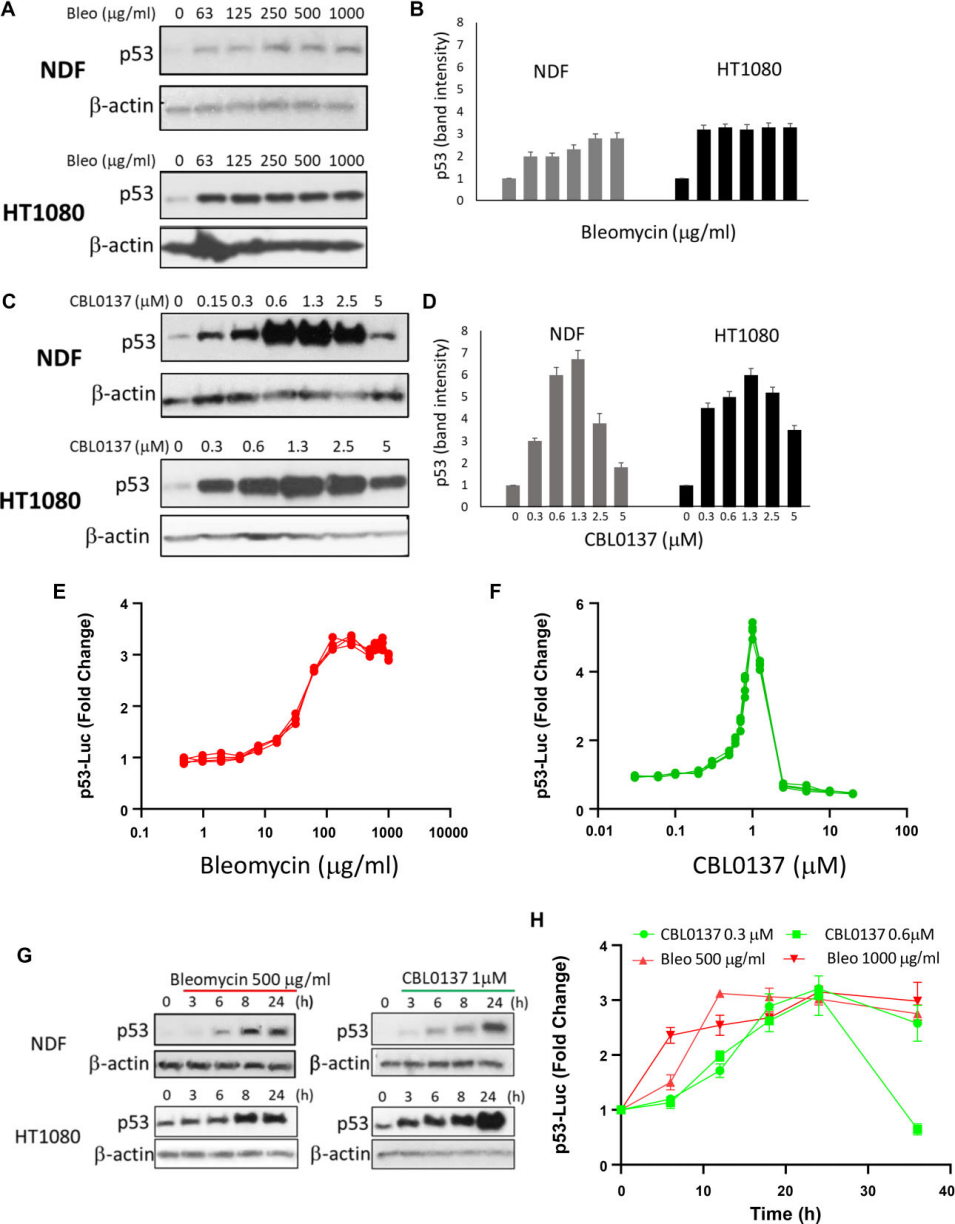
p53 activation by DnaD and ChrD. (A–D) Western blotting of lysates from NDFs and HT1080 cells treated with different concentrations of bleomycin (bleo) (**A, B**) or CBL0137 (**C, D**) for 24 h. (A, C) Images of representative experiments. (B, D) Quantitation of A and C. Bars—mean of two experiments, error bars - SDV. E, F. Induction of p53-responsive luciferase reporter in HT1080 cells treated with different concentrations of bleomycin (**E**) or CBL0137 (**F**) for 24 h. Three replicates for each drug are shown. (**G**) Western blotting of lysates from NDFs and HT1080 cells treated with 500 μg/ml of bleomycin or 1 μM CBL0137 for the indicated times. (**H**) Activation of p53-responsive luciferase reporter in HT1080 cells treated with two concentrations of each drug for the different times.

An important common feature of DnaD and ChrD is the activation of p53 (6,45). We have shown before and confirmed here that p53 accumulated in response to ChrD lacked posttranslational modifications typical for DnaD, e.g. phosphorylation of serine 15 of p53 (Supplementary Figure S1F, G) (6). Here, we observed that the dynamics of p53 protein accumulation differed between DnaD and ChrD. Bleomycin treatment increased p53 protein level starting from the lowest concentration used (63 μg/ml) with weak (NDF cells) or no (HT1080) further increase until the highest concentration (1000 μg/ml) (Figure 2 A, B), whereas CBL0137 induced a sharp increase in p53 protein levels in both cell types with a peak between 0.6 and 1.2 μM followed by a decrease (Figure 2C, D). In both cell types p53 was accumulated to higher levels in response to ChrD than to DnaD (Figure 2A–D).

To confirm that unmodified p53 accumulated upon ChrD is transcriptionally active, we used HT1080 cells with genome-integrated luciferase reporter controlled by a p53 consensus element and p53 binding site from the *CDKN1A* promoter (27,46). In line with p53 protein accumulation, p53 transcriptional activity had different dynamics in response to DnaD and ChrD agents. The p53-responsive transcription quickly reached a plateau and remained at the same level over a wide range of bleomycin concentrations (Figure 2E). The CBL0137 dose-response curve for p53 transcriptional activity was bell-shaped with a narrow peak at approximately 0.5–1.5 μM (Figure 2F). Bell-shaped curves of p53 accumulation and activation in response to CBL0137 may be explained by the inhibition of general transcription by higher doses of the drug (unpublished data).

The kinetics of the p53 protein increase was similar between the two compounds and cell types (Figure 2G). The kinetics for p53 transactivation paralleled that of p53 accumulation for both agents (Figure 2H). Thus, DnaD caused less intensive but steady state p53 activation at a wide concentration range, whereas p53 accumulation in response to ChrD was higher but limited to a narrow concentration range.

### Chromatin damage induces broader changes in gene expression than DNA damage

To compare the transcriptional response of cells to DnaD and ChrD, we selected the lowest concentrations of both agents that caused maximal p53 accumulation and reporter activation 24 h after the start of treatment (500 μg/ml bleomycin and 1 μM CBL0137). To separate p53-dependent and -independent changes in gene expression, we deleted the p53 gene from HT1080 cells and NDFs using the CRISPR/Cas9 approach. To avoid potential DnaD from constitutive Cas9 expression in cells, we transfected cells via electroporation with recombinant Cas9 protein and reconstituted the CRISPR ribonucleoprotein complex with four synthetic gRNAs targeting the first and the last exons of p53. Normally, this approach requires cell cloning and sequencing of target regions to identify the biallelic deletion. However, we used single-cell RNA sequencing (scRNA-seq) of the whole cell population after electroporation to avoid potential artifacts associated with clonal variability (see details in Material and Methods). This method allowed us to distinguish cells with and without functional p53 by their transcriptome, and we determined the proportion of cells that lost p53 after electroporation by p53 staining (Supplementary Figure S7). Subsequent experiments were performed with cell populations consisting of an approximately 1:1 ratio of p53 negative and p53 positive cells. Labeling cells treated under different conditions (control, CBL0137 and bleomycin) with different oligonucleotide-conjugated antibodies allowed library preparation and sequencing of all samples simultaneously as one pool for each cell type.

**Figure 3.**
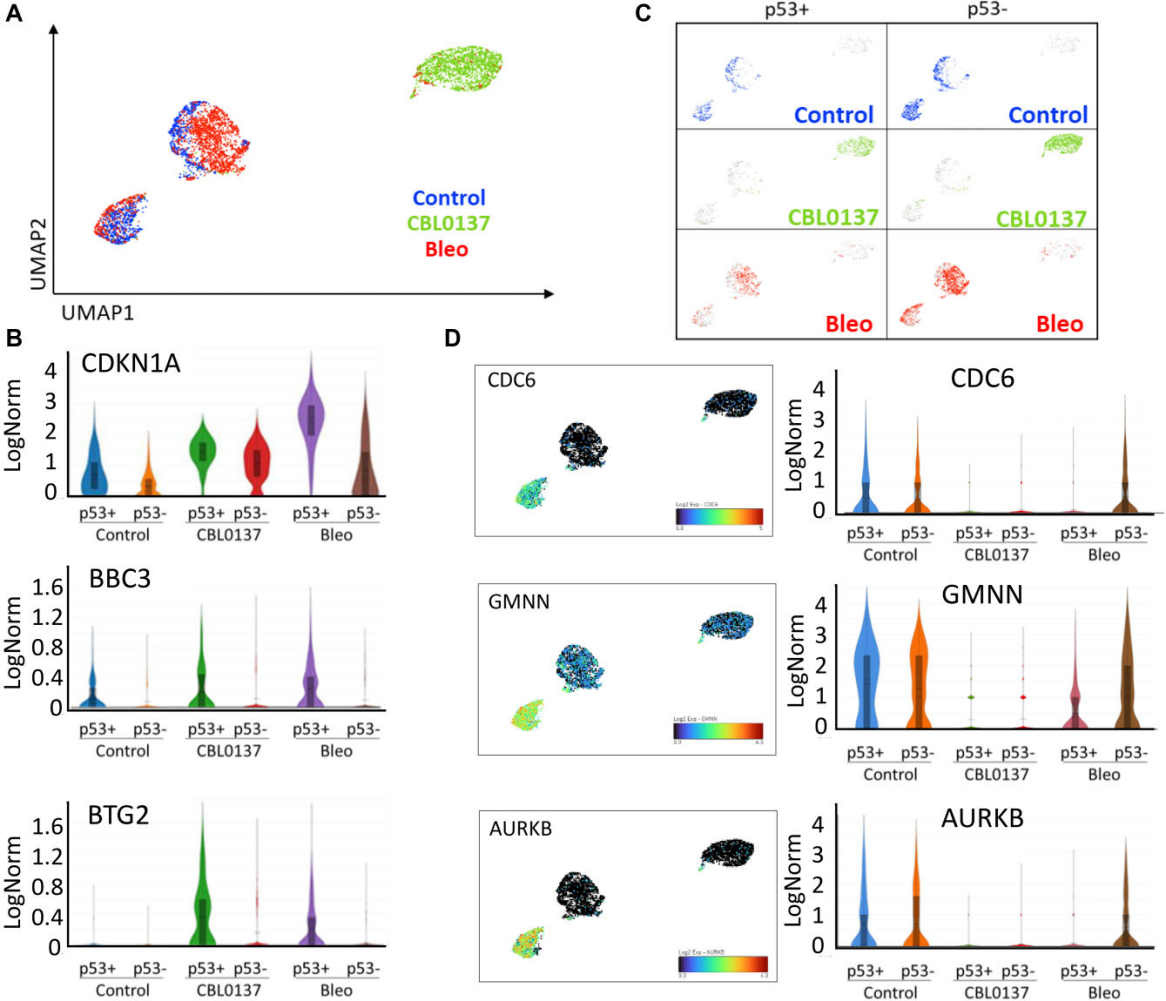
Effects of DnaD and ChrD on cell transcriptome of NDF cells detected by scRNA-seq. (**A**) UMAP plot shows the positions of– control (blue), bleomycin (bleo)-treated (red), or CBL0137-treated (green) cells. (**B**) Violin plots showing the distribution of expression of p53 regulated genes under different conditions and between cells classified as p53-positive or p53-negative. (**C**) UMAP plots of NDF cells color-coded based on the treatment condition (as in A) and p53 status. (**D**) UMAP and violin plots showing the expression of markers for the G1, S and G2/M phases of the cell cycle in NDFs cells under different conditions.

**Figure 4.**
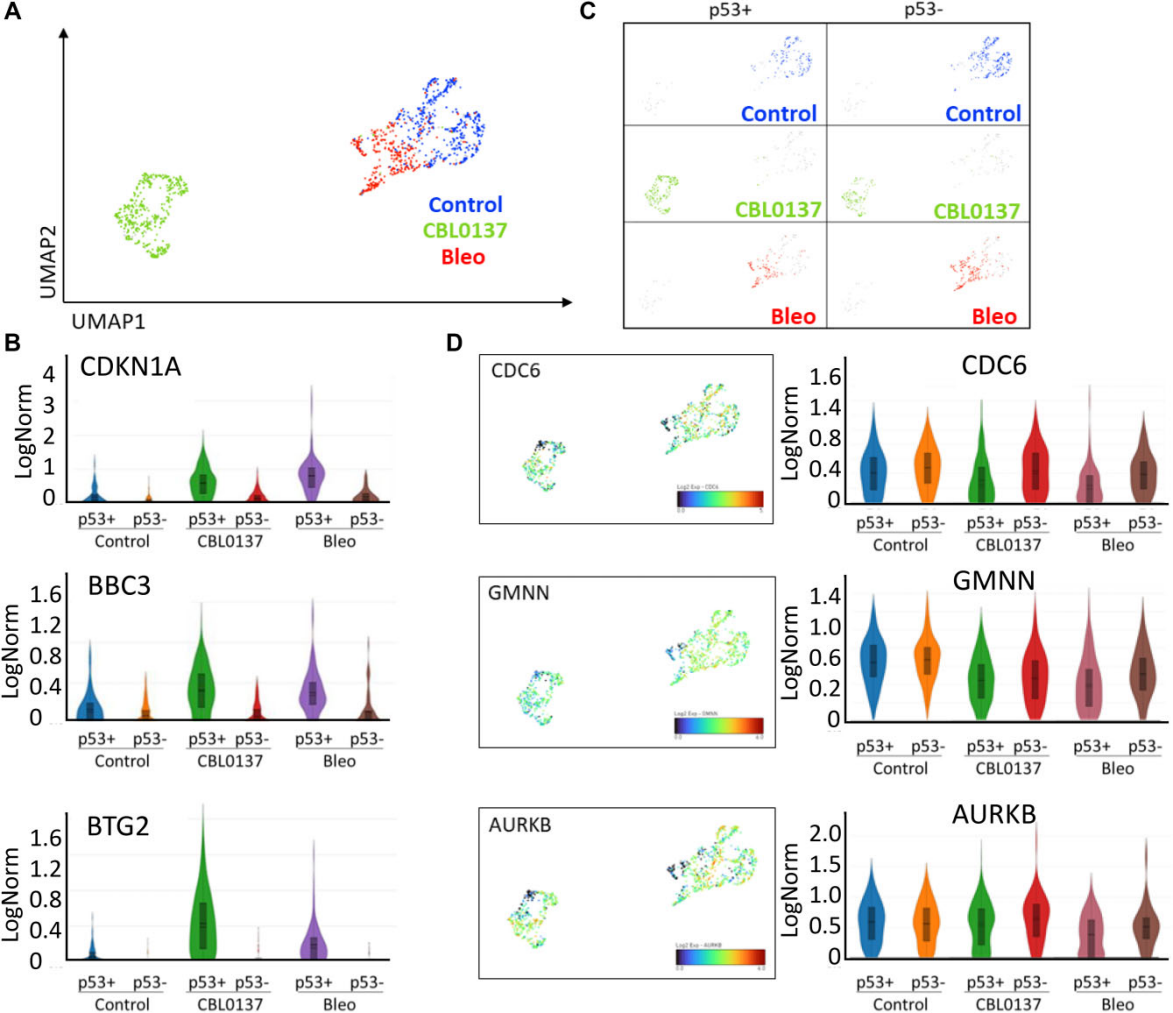
Effects of DnaD and ChrD on cell transcriptome of HT1080 cells detected by scRNA-seq. (**A**) UMAP plot shows the positions of– control (blue), bleomycin (bleo)-treated (red), or CBL0137-treated (green) cells. (**B**) Violin plots showing the distribution of expression of p53 regulated genes under different conditions and between cells classified as p53-positive or p53-negative. (**C**) UMAP plots of HT1080 cells color-coded based on the treatment condition (as in A) and p53 status. (**D**) UMAP and violin plots showing the expression of markers for the G1, S and G2/M phases of the cell cycle in HT1080 cells under different conditions.

Unbiased clustering and UMAP plots demonstrated that both normal and tumor cells formed a diagonal axis along UMAP1 and UMAP2 with groups of control and CBL0137-treated cells located most distantly from each other and a group of bleomycin-treated cells located between those two groups and closer to the control (Figures 3A and 4A), suggesting that ChrD has stronger effects on the cell transcriptome than DnaD.

To identify p53-positive and p53-negative cells, we first looked for the presence of reads corresponding to *TP53*; however, most cells had very low numbers of *TP53* reads (0–3) independent of cell type or treatment. Therefore, we also used reads corresponding to the most specific p53 target, *MDM2* (Supplementary Figure S8A–H, details are in Materials and Methods) to distinguish p53 positive and negative cells. Other p53 targets showed expression patterns similar to MDM2 (Figures 3B and 4B). Surprisingly, this analysis showed that p53-positive and p53-negative cells were located within the same overlapping clusters on the UMAP plots in the case of the control and CBL0137-treated cells or very close in the case of bleomycin-treated cells (Figures 3C and 4C). Thus, although p53-positive and p53-negative cells could be distinguished by the expression of several p53 target genes, the p53 status had a weak impact on the cell transcriptome in response to DnaD and almost no impact in response to ChrD or under unstressed conditions.

In case of NDFs, untreated and bleomycin-treated cells were located close to each other on the UMAP plots, although they did not overlap (Figure 3A). These cells were found within two clusters: one mostly consisting of untreated cells (Figure 3A, left cluster) with a small admixture of bleomycin-treated cells, and the second cluster was comprised predominantly of bleomycin-treated cells with an ‘edge’ of untreated cells (Figure 3A, central cluster). A major difference between these two clusters was in the expression of cell cycle markers, including genes predominantly transcribed during G1 (*CDC6*, *CDC25B*), S (*CDC45, MCM4, EXO1, GMMN1*), and G2/M (*AURKB, MKI67*) phases of cell cycle (Figure 3D, Supplementary Figure S8I). The left cluster was positive for these markers and consisted of control cells and p53-negative bleomycin-treated cells. The central cluster negative for these cell cycle-associated markers contained p53-positive bleomycin-treated cells and non-cycling control cells (Figures 3A, C, Supplementary Figure S8I). These results suggest that the major response of normal fibroblasts to DnaD is p53-dependent growth arrest. Moreover, p53-positive bleomycin-treated cells were not significantly different from non-cycling control cells, reinforcing the concept that normal fibroblasts respond to DnaD with growth arrest.

Control and bleomycin treated HT1080 cells occupied two adjacent regions within one cluster (Figure 4A). The cells within this cluster formed a gradient transition from high to low expression of cell cycle markers and from low to high p53 positivity without clear boundary between samples (Figure 4A, D and Supplementary Figure S8F–H, J). This gradient started from the untreated cells (the highest cell cycle marker levels) followed by p53-negative bleomycin-treated cells (high cell cycle markers) and then p53-positive bleomycin-treated cells (lower cell cycle marker levels). The difference between the expression levels of the cell cycle markers was less pronounced in tumor than normal cells (compare Figures 3D versus 4D and Supplementary Figure S8I versus S8J), suggesting that in contrast to normal cells, there is more variability in the tumor cell response to DnaD with a less pronounced cell cycle arrest and less dependence on p53.

**Figure 5.**
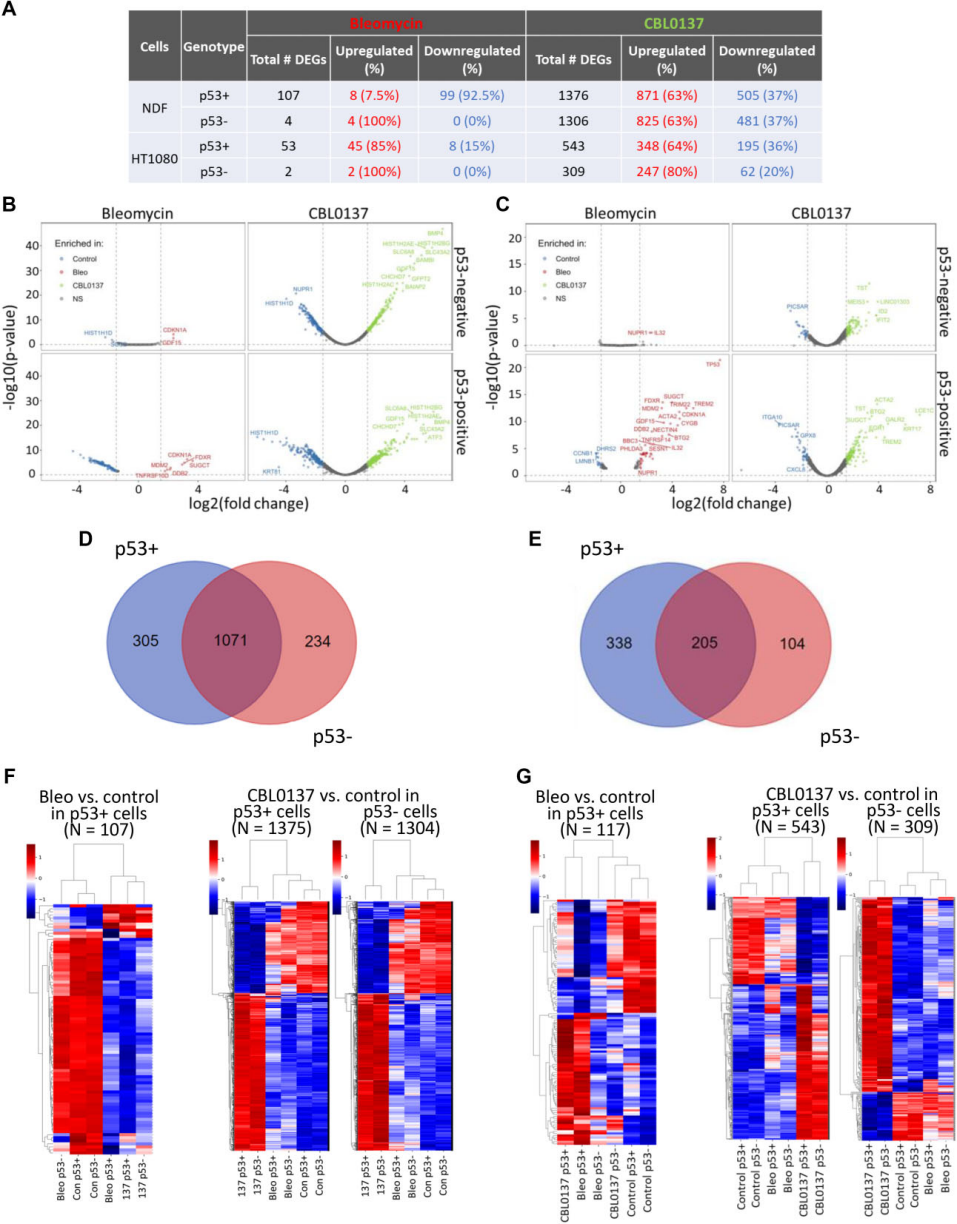
Transcriptional response to ChrD is p53-independent. (**A**) Number of differentially expressed genes (DEGs, FC > 1.5, adjusted *P*-value < 0.05) between different treatment conditions in p53-positive and p53-negative cells. (B, C) Volcano plots showing the DEGs in p53-positive and p53-negative NDFs (**B**) and HT1080 cells (**C**) under different treatment conditions (control [Con], blue; bleomycin [Bleo], red; CBL0137, green). (D, E) Venn diagram showing the overlap of DEGs in p53-positive and p53-negative NDFs (**D**) and HT1080 cells (**E**). (F, G) Dendrograms and heatmaps showing unbiased hieratical clustering of samples and genes in NDFs (**F**) and HT1080 cells (**G**). The number of DEGs (N) with *P*_adj_ < 0.05 is indicated at the top of each plot. No plots were built from the DEGs of p53-negative bleomycin-treated cells due to the low number of DEGs (see panel A).

CBL0137-treated cells were clearly separated from the other samples (Figures 3A and 4A), consistent with many more genes which expression has been changed in response to ChrD than DnaD: CBL0137 treatment altered the expression of 1375 genes in p53-positive NDFs, 1304 genes in p53-negative NDFs, 543 genes in p53-positive HT1080 cells, and 309 genes in p53-negative HT1080 cells (*P*_adj_ < 0.05) (Figure 5A). In contrast, bleomycin treatment changed the expression of only 107 genes in p53-positive NDFs, 4 genes in p53-negative NDFs, 117 genes in p53-positive HT1080 cells and 2 genes in p53-negative HT1080 cells (Figure 5A). Thus, ChrD caused much more dramatic changes in the cell transcriptome than DnaD. Moreover, there was a striking p53 dependence in response to DnaD, whereas ChrD caused a transcriptional response with a magnitude indicative of a p53-independent response (Figure 5A–C). Furthermore, most genes whose expression changed in response to ChrD were the same in the p53-positive and p53-negative cells (Figure 5D–G), further supporting the hypothesis that the transcriptional response to ChrD is largely p53-independent.

### Gene expression changes in response to DNA damage reflect p53-dependent growth arrest

**Figure 6.**
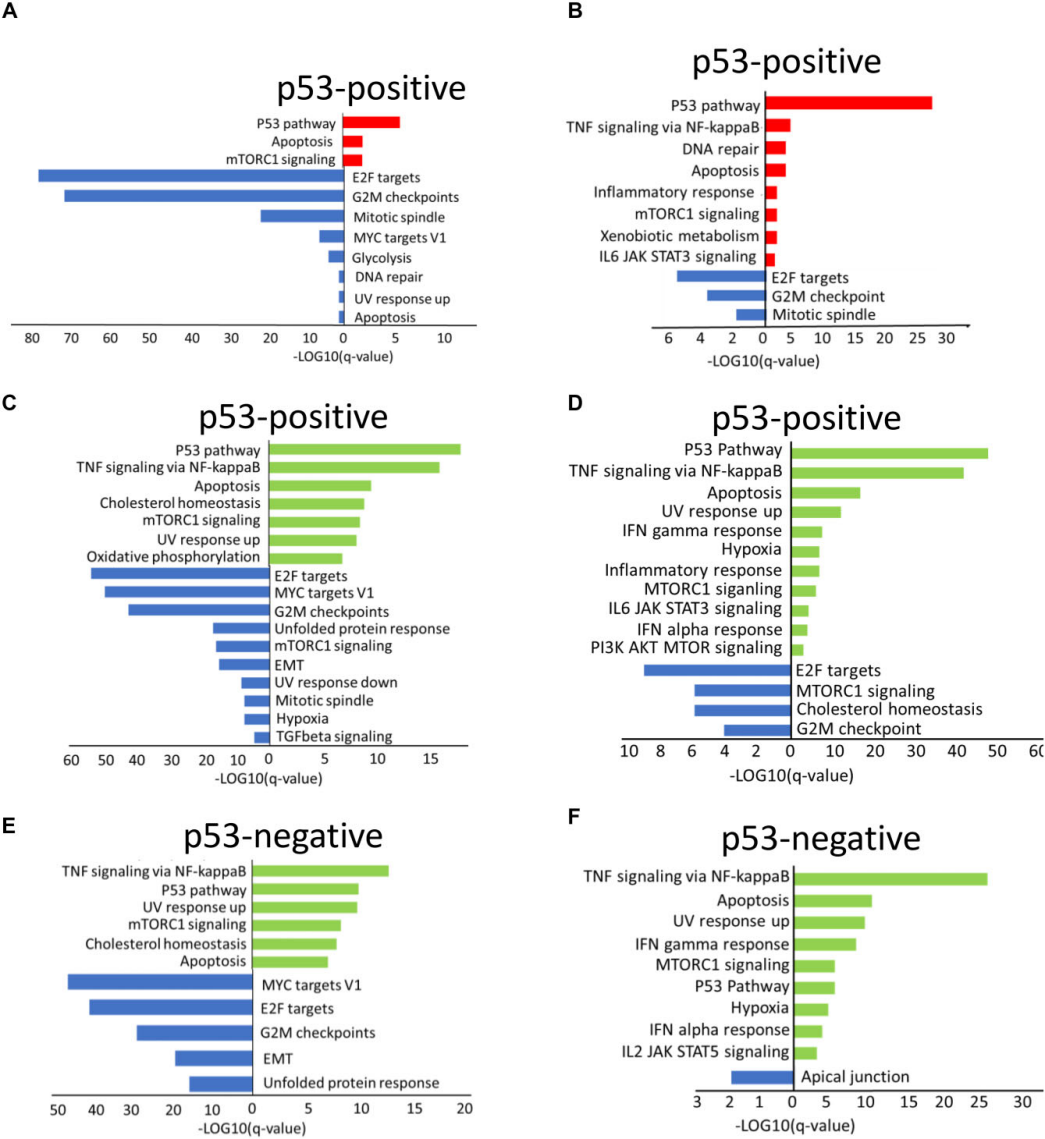
Gene set enrichment analysis of differentially expressed genes between different conditions identified by scRNA-seq in NDFs (**A**, **C**, **E**) and HT1080 cells (**B**, **D**, **F**). (A, B) Hallmark gene sets enriched in control (blue) or bleomycin-treated (red) p53-positive cells. (C, D) Hallmark gene sets enriched in control (blue) or CBL0137-treated (green) p53-positive cells. (E, F) Hallmark gene sets enriched in control (blue) or CBL0137-treated (green) p53-negative cells.

The transcriptional response to bleomycin was strikingly different between the two p53 genotypes. Although the expression levels of a substantial number of genes were changed in the p53-positive NDFs (107 genes) and HT1080 cells (53 genes) following treatment with bleomycin, the expression of only four genes in the NDFs and two genes in the HT1080 cells were changed in response to the drug in p53-negative cells, demonstrating the major role of p53 in the DnaD response (Figure 5A–C). Only 8 out of 107 changed genes were upregulated in the NDFs, among which four were p53 targets (*CDKN1A, MDM2, DDB2* and *FDXR*). The 99 suppressed genes were highly enriched for ‘E2F targets’ and ‘G2M checkpoint’ (Figure 6A) and represented almost exclusively genes expressed during the cell cycle, indicating that these cells were growth arrested. The ratio between the upregulated and downregulated genes was reversed for the tumor cells: 45 genes were upregulated, and eight were downregulated. The upregulated genes were strongly enriched for ‘p53 targets’ (18 out of 45), whereas the downregulated genes were enriched for ‘E2F targets’ and ‘G2M checkpoint’ (6 of 8 combined) (Figure 6B). Thus, the response to DnaD in these cells was almost exclusively through p53 and included p53-dependent growth arrest, which was stronger in normal cells than in tumor cells.

### Minimal effect of p53 on the transcriptome of control and CBL0137-treated cells

To look more carefully at the influence of p53 on transcription, we compared gene expression in p53- positive and p53-negative cells under different conditions. Only four genes in the NDFs (*TP53, MDM2, CDKN1A* and *GDF15*) and seven in the HT1080 cells (*TP53, MDM2, CDKN1A, GDF15, FN1, ZMAT3* and *FDXR*) two of which were used for cell classification, varied between untreated p53-positive and p53-negative cells. The expression levels of all these genes were higher in p53-positive cells. The small difference between p53 positive and negative control cells suggested that p53 had a minimal effect on gene expression in unstressed conditions in line with very low mRNA and protein levels. Surprisingly, a similar situation was observed in CBL0137-treated cells: 12 genes in the NDFs and 43 genes in the HT1080 cells were significantly higher in p53-positive versus negative cells, and no genes were significantly lower. Although there were p53 targets among the genes expressed higher in CBL0137-treated p53-positive cells than in CBL0137-treated p53-negative cells (3 out of 12 genes in NDF and 20 out of 43 in HT1080), this low number of genes differing between genotypes relative to the total number of differentially expressed genes in response to CBL0137 (Figure 5A), confirms that the ChrD response is primarily p53-independent.

There were consistently more genes upregulated than downregulated in response to CBL0137 in both genotypes and cell types (Figure 5). Genes downregulated in CBL0137-treated cells were also enriched for ‘E2F targets’ and the ‘G2M checkpoint’ (Figure 6C–F). Upregulated genes mainly belonged to two lists, ‘p53 targets’ and ‘TNF signaling via NF-kappaB'. These lists were enriched within both genotypes (Figure 6C–F).

To confirm that the response to ChrD is largely p53-independent, we used additional methods of p53 inactivation and assessment of transcription. We inactivated p53 in HT1080 cells using the dominant-negative mutant GSE56 that prevents p53 oligomerization (28) and then sequenced newly synthesized transcripts labeled with EU for 20 min at the end of the 24 h treatment. Newly synthesized EU labeled RNA was purified and used for nascent RNA-seq. As expected, bleomycin did not induce p53 targets in the cells with inactive p53; however, there was almost no difference in the responses of p53 target genes to CBL0137 between cells with active or inactive p53 (Supplementary Figure S9).

Our experiments clearly demonstrated different changes in the cell transcriptome in response to DnaD and ChrD. There were many more genes which expression was changed in response to ChrD than DnaD. While response to DnaD was highly p53 dependent, response to ChrD was largely p53 independent. At the same time, ChrD induced the expression of p53-dependent genes. The biggest puzzle is that these ‘p53-dependent’ genes were also induced in p53-negative cells.

### Genes activated by ChrD have a unique chromatin state at their promoters

**Figure 7.**
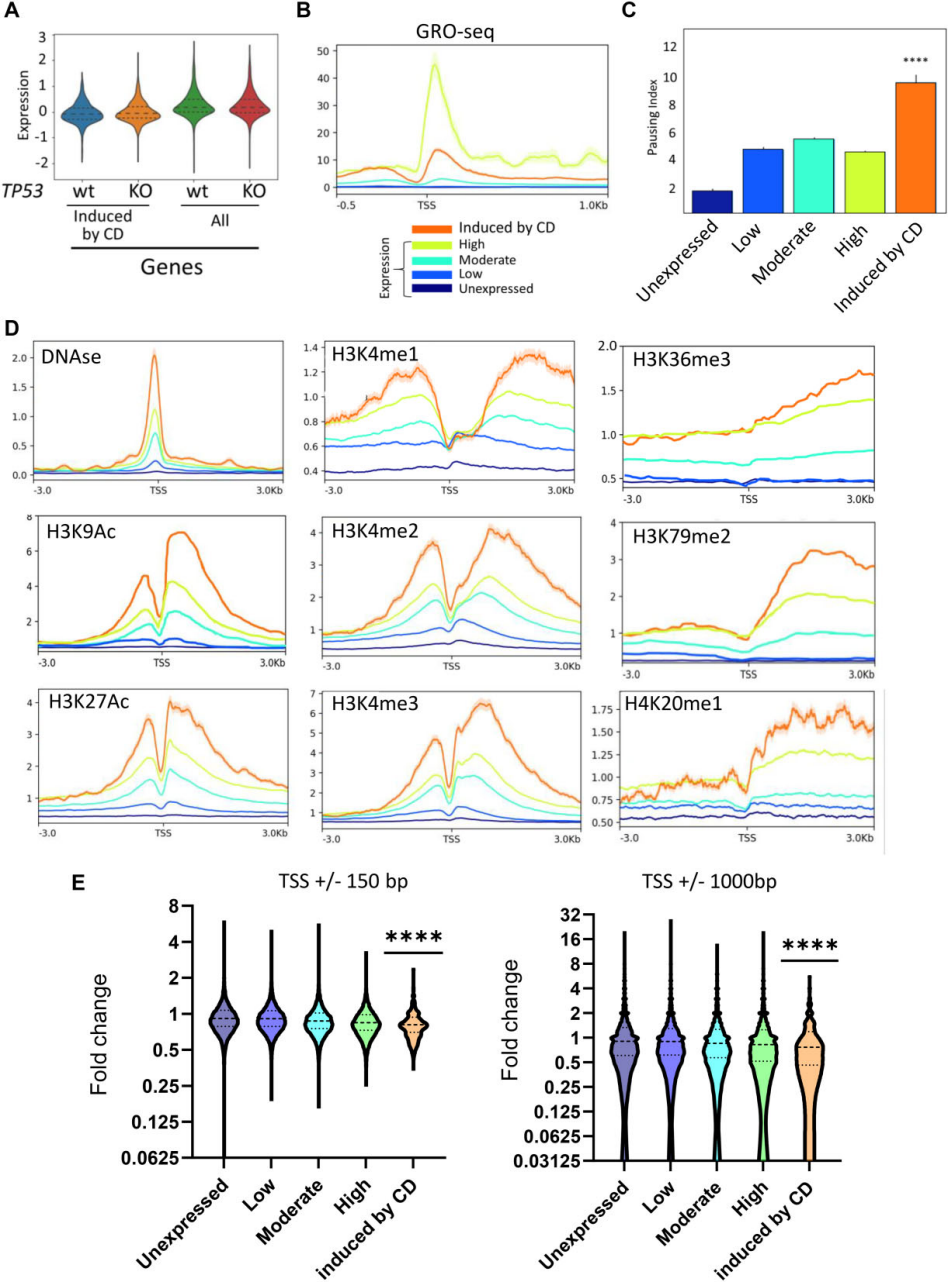
Genes activated by ChrD have a specific chromatin state around TSS. (**A**) Comparison of expression levels of genes activated by CBL0137 in p53-positive and p53-negative NDFs with the expression levels of all other genes. Violin plots of normalized expression levels obtained by bulk RNA seq. (**B**) Normalized profiles of GRO-seq for different categories of genes in NDFs. (**C**) RNA polymerase II pausing index calculated using the GRO-seq data of IMR90 cells. ****P < 0.0001 by the Kruskal–Wallis test. (**D**) Metagene profiles of DNAse hypersensitivity (DNAse) and the indicated histone post-translational modifications for different categories of genes. The same legend as in panel B. (**E**) Change in MNase-seq read density at the indicated distances around the TSS of different genes in HT1080 cells treated with 1 μM CBL0137 for 1 h versus the control. ****P < 0.0001 by two-tailed *t*-test, ChrD-induced genes versus other groups (comparison between other groups is not shown).

p53 targets were the most enriched category among genes activated by ChrD in all cells (i.e. normal and tumor, p53-positive and p53-negative). Besides being enriched within the category of ‘p53 target genes’, these genes were also enriched within the ‘TNF signaling via NF-kappaB’ and several other categories that collectively could be characterized as ‘stress-response genes’ (Figure 6C–F). Several reports have shown that many stress-response genes are regulated at the level of RNA polymerase II pausing (47,48). Under basal conditions, these genes have preassembled RNA polymerase II at their promoters engaged in abortive elongation until a specific transcription factor binds and releases RNA polymerase II into productive elongation through a currently undefined mechanism (49). Thus, we hypothesized that stress-response genes induced by CBL0137 might have a specific chromatin state around their TSS, making them sensitive to ChrD. Thus, we examined the state of chromatin around the TSS of genes activated by ChrD in both p53-positive and p53-negative cells under basal conditions (i.e. untreated) using our data and publicly available datasets. More data was available for NDFs, although the limited data for HT1080 cells confirmed our finding in the NDFs. We compared genes induced by ChrD with all other genes classified by their expression levels under basal conditions based on bulk (NDFs) or nascent (HT1080) RNA sequencing. Genes were divided into four quartiles: (i) no expression, (ii) low expression, (iii) moderate expression, and (iv) high expression levels. Analysis of the publicly available GRO-seq data for IMR90 cells (human lung fibroblasts, (50)) confirmed this classification (Figure 7A, B). Based on the average expression of ChrD-induced genes under basal conditions, these genes were in between the highly and moderately expressed genes (Figure 7B).

We looked at RNA polymerase II pausing in these quartiles using the IMR90 GRO-seq dataset. Consistent with our hypothesis, ChrD-induced genes had the highest pausing index in basal conditions (Figure 7C). Next, we evaluated DNAse hypersensitivity and histone marks associated with active and repressed states. DNA around the TSS of ChrD-induced genes was more sensitive to nuclease digestion than DNA of the most highly expressed genes (Figure 7D), suggesting the presence of nucleosome-depleted regions. Moreover, the nucleosomes present around the TSS of these genes had the highest levels of activating histone marks, including H3K27Ac, H3K9Ac, and mono-, di-, and trimethylated H3K4 (Figure 7D). Interestingly, these genes also had the highest levels of histone marks associated with active elongation downstream of their TSS, such as H3K36me3, H3K79me2 and H4K20me1 (Figure 7D). Repressive histone marks around TSS of ChrD-induced genes were similar to these marks at highly expressed genes (Supplementary Figure S10A).

These analyses showed that genes activated by ChrD have a specific chromatin state with a high level of paused RNA polymerase II. ChrD may activate transcription of these genes either because nucleosomes in this state are more sensitive to ChrD than other nucleosomes or because CBL0137 triggers other mechanisms that allow the release of paused RNA polymerase II. To distinguish between these hypotheses, we measured nucleosome loss from the same categories of genes upon treatment with CBL0137 using micrococcal nuclease (MNase) digestion followed by sequencing (MNase-seq) (32). Indeed, we found that on average, ChrD-induced genes lost more nucleosomes at and around TSS than genes from other categories (Figure 7E and Supplementary Figure S10B).

Although the genes activated by ChrD did not fall within the quartile of the most highly expressed genes, they had a chromatin composition at and around their TSS that was more open and more decorated with the histone marks for active transcription than the most highly expressed genes. Their gene bodies contained more histone marks of active elongation than the most actively transcribed genes. These genes were enriched within different stress-response categories and had the highest RNA polymerase II pausing index. Nucleosomes at regions around TSS of these genes are also the most sensitive to nucleosome destabilizing effect of ChrD.

### Chromatin damage activates a p53-regulated reporter in the absence of p53

**Figure 8.**
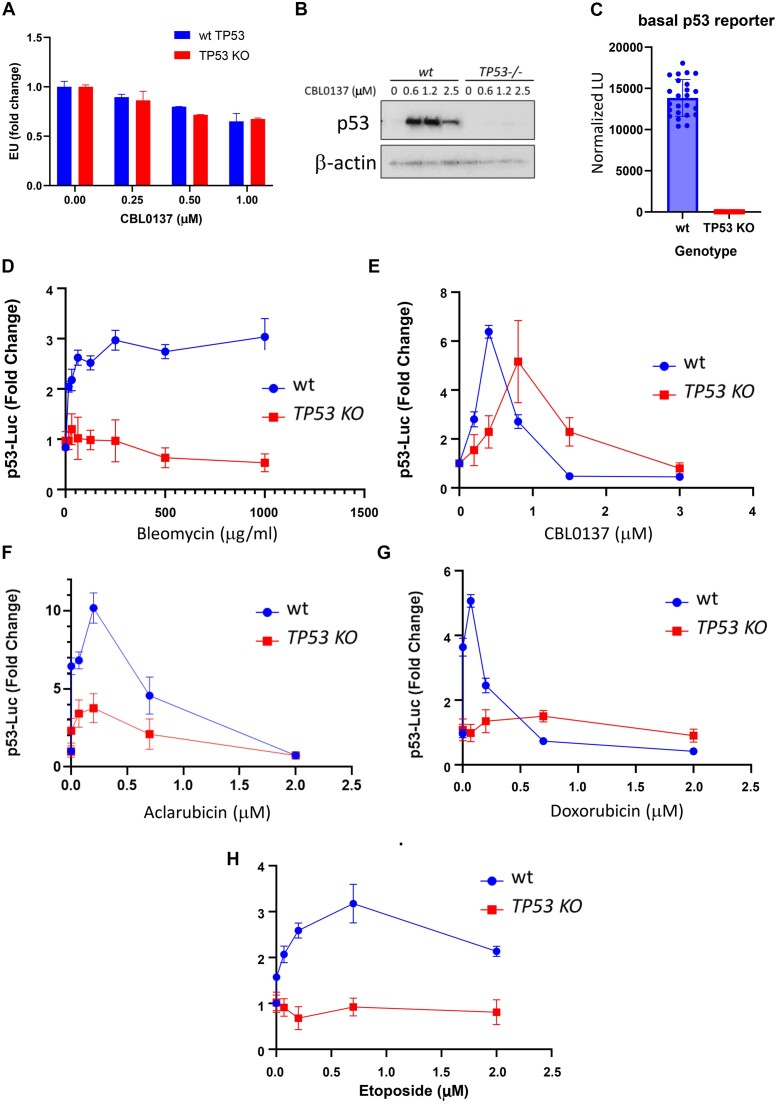
ChrD, but not DnaD, induces p53 transcriptional activity in the absence of p53. (**A**) Effect of CBL0137 on general transcription. EU incorporation assay for p53-positive and p53-negative HT1080 cells treated with CBL0137 for 3 h. Data are presented as the mean ± SD (*n* = 2 replicates). (**B**) Western blotting of lysates from p53-positive and p53-negative HT1080 cells treated with CBL0137 for 24 h. (**C**) Basal luciferase activity controlled by a p53-responsive promoter in p53-positive and p53-negative HT1080 cells, normalized by cell number. Data are presented as the mean ± SD (*n* = 6 replicates in three experiments). (D–H) Fold-change in p53-responsive luciferase activity in p53-positive and p53-negative HT1080 cells treated with bleomycin (**D**), CBL0137 (**E**), aclarubicin (**F**), doxorubicin (**G**) or etoposide (**H**) for 24 h.

Higher nucleosome loss at promoters of ChrD-induced genes suggests that ChrD may trigger transcription of these genes in the absence of specific transcriptional factor through the direct effect on chromatin. This hypothesis is in line with similar degree of activation of p53 responsive genes in p53 positive and negative cells. Thus, we decided to test whether ChrD can induce activity of a specific p53 reporter in the absence of p53. First, we excluded that CBL0137 activated general transcription using the EU incorporation assay (Figure 8A). Then we transduced p53-positive and p53-negative HT1080 cells with lentivirus harboring p53-responsive luciferase reporter construct.(Figure 8B). In untreated condition, reporter activity was >500 times higher in the p53-wild type than in the knockout cells (Figure 8C). This was surprising, taking into account the very low number of p53 transcripts in cells and no major impact of p53 on the cell transcriptome under basal conditions. This suggests that just a few p53 molecules in some cells are enough to cause a transcriptional burst; however, this burst involves only a few genes and does not significantly affect cell transcriptional programs.

When we treated this pair of reporter cells with bleomycin or CBL0137 for 24 h, we observed that DnaD activated the p53-responsive reporter in the p53 wild-type cells but had no effect on the reporter in p53-deficient cells (Figure 8D). In contrast, ChrD activated the reporter regardless of p53 status, demonstrating that ChrD could activate transcription without a specific transcription factor (Figure 8E).

Because this phenomenon was highly unusual, we tested other chemicals differing in their ability to cause ChrD or DnaD in the same pair of reporter cell lines – two anthracyclines (doxorubicin and aclarubicin) and the podophyllotoxin etoposide. The two anthracyclines differ in their ability to cause DnaD or ChrD. Doxorubicin causes DnaD via direct chemical reaction with DNA (51). It also causes ChrD, which is weaker than ChrD from CBL0137 or aclarubicin (11). Aclarubicin causes weaker DnaD than doxorubicin but induces ChrD comparable to CBL0137 (11). Etoposide, a non-DNA-binding topoisomerase II inhibitor, causes DnaD but no ChrD (11). Both anthracyclines activated the p53 reporter in the absence of p53; the degree of activation corresponded to their ChrD activity (Figure 8F, G). Like bleomycin, etoposide did not activate the p53-responsive reporter in the p53-negative cells (Figure 8H).

We tested the response of p53 reporter in several additional cell lines differing in p53 status, wild type (HCT8, MCF7) of mutant (HT29 – p53^237R^). We generated these reporter cell lines for prior studies several years ago. p53-reporter in these cells was almost silenced independently on their p53 status and was barely detectable in basal conditions (Supplementary Figure S11A). When we treated these cells with bleomycin, p53 reporter was activated only in p53 wild type cells (Supplementary Figure S11F), while all compounds which had ChrD activity, CBL0137, aclarubicin and doxorubicin, activated p53 reporter in wild type and mutant cells (Supplementary Figure S11C–E).

Thus, our data suggest that although DnaD induces p53 targets by activating p53 protein, ChrD can activate these targets without functional p53 protein.

## Discussion

In this study, we identified the commonalities and differences in the response of normal and tumor cells to DnaD and ChrD. Most of our observations were performed shortly after the induction of DnaD by a drug mimicking nuclease activity or ChrD by a drug directly interfering with DNA-histone binding. We selected bleomycin because short-term treatment does not damage nucleosomal DNA, leaving nucleosomes intact (16,21,19,52), and CBL0137 because it does not cause detectable DnaD (5,7). However, we cannot exclude ChrD as a later secondary effect of bleomycin treatment because DNA breaks facilitate spontaneous DNA unwrapping from the histone core and chromatin disassembly during DNA repair. Massive and quick histone loss from chromatin upon bleomycin treatment was shown in yeast (53), however, we did not observe this in mammalian cells during the time of observation. This may be explained by a larger genome and more local chromatin disassembly around sites of DnaD. Fast local chromatin disassembly and reassembly after UV was shown by Adam et al in mammalian cells (54). However, it was shown that in case of chromatin disassembly in response to DnaD, histones are accompanied by histone chaperones (55,56) and this most probably prevents their loss from chromatin as in case of ChrD drugs. Furthermore, DnaD may be a secondary effect of CBL0137 treatment due to a plethora of mechanisms, e.g. DNA unwrapped from the histone core is more exposed to water molecules, increasing the chances of encountering reactive oxygen species, dysregulation of transcription should lead to R-loop formation (57) and transcription replication conflicts (58). Currently we cannot exclude these effects, however, we did not detect DNA damage during the period of observation.

DnaD and ChrD are both cytotoxic; and tumor cells are more sensitive to both types of damage than normal cells. Both types of damage also activate p53. However, both these consequences of DnaD and ChrD have features specific to the type of damage. The major difference in cytotoxicity induced by DnaD and ChrD is that DnaD causes cellular senescence, while ChrD does not. This difference likely explains the recently reported absence of cardiotoxicity from aclarubicin treatment, which causes predominantly ChrD (11). Doxorubicin is structurally similar to aclarubicin but causes strong DnaD due to chemical reaction with DNA (59) and has a strong cardiotoxic effect (17,18). Importantly, anti-cancer activity of both compounds was similar (18), suggesting that anti-cancer efficacy depends on ChrD, while the toxicity on DnaD.

Another interesting feature that distinguishes the toxic effects of DnaD and ChrD is that bleomycin toxicity is almost linearly proportional to the dose administered and possibly the number of DNA breaks, although we did not measure this. In contrast, CBL0137 shifts from non-toxic to fully toxic at a narrow concentration range. This phenomenon may be explained by one of two models: (i) chromatin tolerates the intercalation of small molecules into DNA without destabilizing nucleosomes up to a certain threshold, e.g. if the intercalator first binds to linker DNA; (ii) chromatin is gradually decondensed but cell viability is compromised only after a certain level of decondensation is reached. Mechanisms of toxicity attributed to chromatin decondensation are still obscure.

Similarly, p53 accumulation following ChrD also occurs within a narrow concentration range from no effect to a much higher p53 accumulation than observed after any dose of DnaD agent. This spike in accumulation is followed by the loss of p53 accumulation with further increases in CBL0137 concentration, likely due to the inhibition of general transcription by CBL0137 at doses higher than 1.5–2 μM (unpublished data). In contrast, p53 accumulation is not proportional to the degree of DnaD and stays at the same level at concentration of bleomycin spanning three orders of magnitude. Based on these findings, we propose that p53 transcription and translation are rate-limiting in response to DnaD and rather low. Therefore, even when only a few DNA breaks activate several DnaD-sensitive kinases, these kinases phosphorylate almost all of the newly synthesized p53, and further increases in the number of DNA breaks and activated kinases do not result in additional p53 stabilization.

We do not know exactly how and why p53 protein levels increase in response to ChrD. We previously described FACT-CK2-mediated phosphorylation of p53 on serine 392 (6); however, this phosphorylation cannot explain p53 accumulation. It is also unclear why CK2 phosphorylates p53 after FACT binds to disassembled nucleosomes. Although we cannot exclude other p53 modifications in response to ChrD that we have not been able to detect so far, p53 is likely significantly less modified in response to ChrD than DnaD.

Because DnaD is considered a major cellular stress with multiple sensors and signaling pathways involved, we were surprised that the transcriptional response to DnaD almost exclusively depends on p53. Although multiple studies have analyzed the transcriptional response to DnaD, those studies were performed using bulk RNA-seq that assessed the average response of millions of cells. At the single-cell level, where hundreds to thousands of cells are processed as individual replicates, we found that only the activation of very few classical p53 targets was statistically significant. DnaD inhibited many more genes in a p53-dependent manner; however, all the inhibited genes were expressed in cycling cells, with ‘E2F targets’, and ‘G2/M checkpoint,’ being the main enriched categories. These findings suggest that DnaD causes p53-dependent growth arrest by inducing *CDKN1A*, and the difference in gene expression is a difference between cycling and arrested cells. In support of this concept, the transcriptome of untreated, non-dividing normal fibroblasts is not different from that of p53-positive bleomycin-treated cells. However, we cannot exclude the role of p53 in suppressing the expression of some of these genes. There are still debates about the role of p53 in suppressing gene expression (60).

Transcriptional changes in response to ChrD differ from p53 signaling in response to DnaD. First, more genes are activated by ChrD than DnaD, and these genes are similarly activated in p53 wild-type and null cells. Moreover, a highly p53-specific reporter was activated by ChrD in the absence of functional p53. Thus, ChrD may act directly on the nucleosomes around the TSS of these genes, facilitating acts of transcription due to the presence of paused RNA polymerase II and a chromatin state characteristic of active transcription.

In p53 wild-type cells, we are most likely observing the combined effects of ChrD directly on nucleosomes or paused RNA polymerase II around the TSS of some genes and the accumulation of high levels of unmodified or weakly modified p53. Experiments using lentiviral delivery of p53 or inducible p53 expression demonstrated that poorly modified p53 is transcriptionally active (27,46). However, the input of p53 protein in the transcriptional response to ChrD does not seem essential because the degree of activation of endogenous p53 targets was not significantly different between p53-positive and p53-negative cells based on nascent RNA-seq data.

In summary, we identified several consequences of ChrD and compared them with those of DnaD. Although both DnaD and ChrD are cytotoxic, there are important differences in their effects in cells, such as the inability of ChrD to induce senescence. Moreover, both DnaD and ChrD are potent activators of p53 and p53-dependent transcription; however, only the response to DnaD is highly p53-dependent. The ChrD response is largely p53 independent. Based on our findings, we propose the following model: ChrD directly affects the promoters of stress-responsive genes, including p53 targets. Genes activated by ChrD have open chromatin and paused RNA polymerase II under basal conditions. Treatment with ChrD agents triggers the transcription of these genes even in the absence of specific transcription factors, such as p53.

## Supplementary Material

gkad865_Supplemental_File

## Data Availability

The following datasets are available at NCBI GEO as GSE223327 and as Sequence Read Archive (SRA) data under project name PRJNA925823: scRNA-seq of NDF and HT1080 cells, untreated or treated with CBL0137 or bleomycin, nascent RNA-seq of HT1080 cells untreated or treated with CBL0137 or bleomycin, MNase-seq data of HT1080 cells, untreated or treated with CBL0137.
