# Correction to ‘Comparison of cell response to chromatin and DNA damage’

**DOI:** 10.1093/nar/gkad1192

**Published:** 2023-12-07

*Nucleic Acids Research*, Volume 51, Issue 21, 27 November 2023, Pages 11836–11855, https://doi.org/10.1093/nar/gkad865

In the originally published version of this manuscript, there was an error in the Funding section, whereby the grant number for author Omar Kantidze was given as RSF [19-74-10018 to OK], instead of RSF [21-74-10018 to OK].

This has been corrected online.

