# Supplementary material for: Comparison of cell response to chromatin and DNA damage
Source: Nucleic Acids Res. 2023 Oct 19;51(21):11836–55. doi: 10.1093/nar/gkad865 (PMC10681726; doi:10.1093/nar/gkad865)
Supplement: gkad865_Supplemental_File [file gkad865_supplemental_file.pdf]

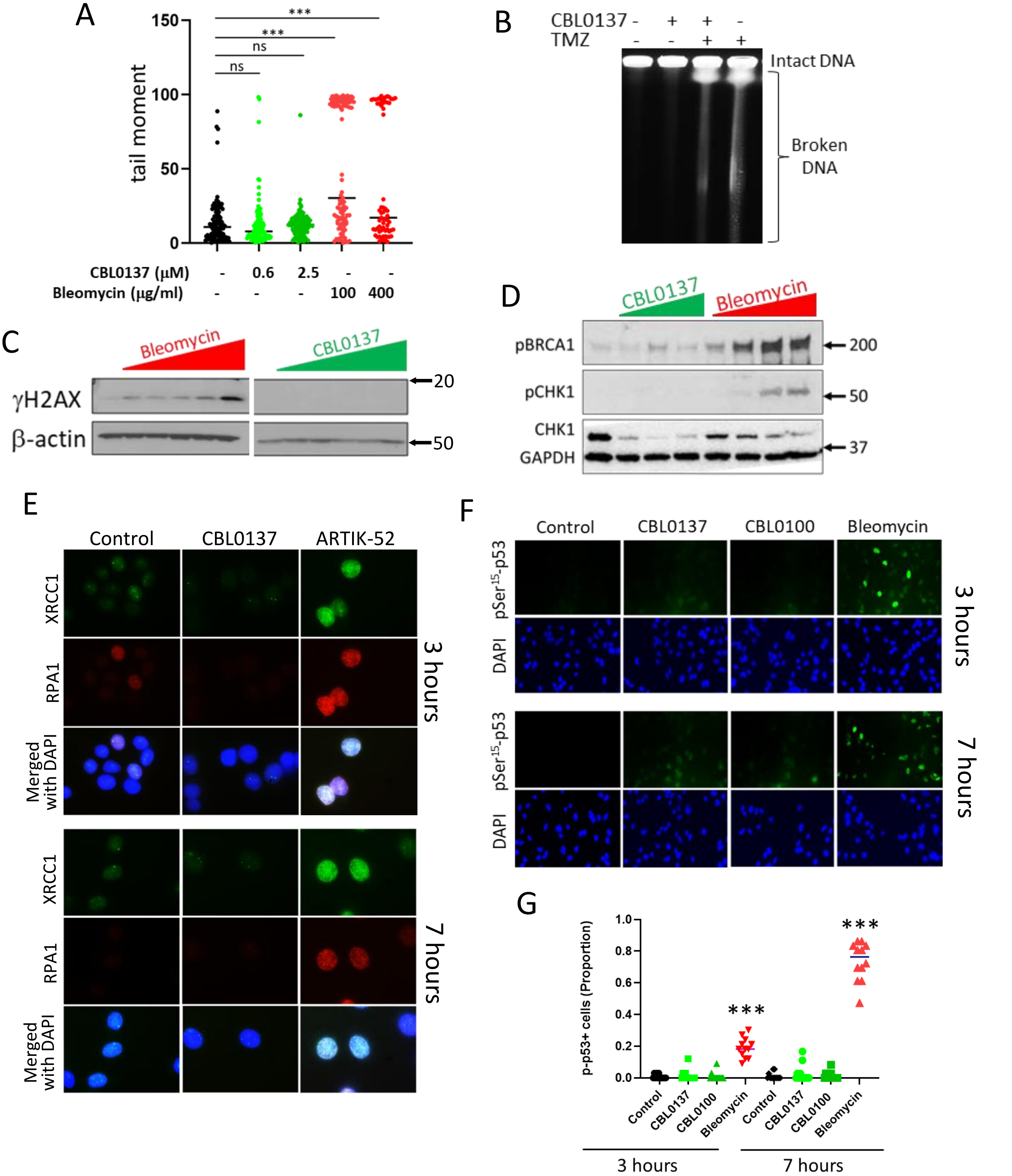

Supplementary Figure S1. Comparison of DNA damage caused by different molecules. A. Alkali comet assay for cells treated with CBL0137 or bleomycin for 24 hours. The tail moment was assessed using the OpenComet plugin of ImageJ. A total of 100 randomly selected cells were evaluated. ***$p < 0.005$ by ANOVA. B. Pulse field gel electrophoresis of BE2C neuroblastoma cells treated with 0.2 µM of CBL0137 or 250 µM of temozolomide (TMZ) for 6 hours. *Continued on the next page.*

Supplementary Figure S1. Comparison of DNA damage caused by different molecules. C, D. Western blotting of protein extracts from HT1080 cells treated with CL0137 or bleomycin for 24 hrs and stained with the antibodies against the indicated proteins. The numbers to the right of the western blots are the positions of the protein size markers in kDa. Treatments: in C- bleomycin was used at 63, 125, 250, 500, and 1000 μg/ml, and CBL0137 was evaluated at 0.325, 0.63, 1.25, 2.5, and 5 μM, in D - bleomycin was used at 63, 125, 250, 500 μg/ml, and CBL0137 at 0. 25, 0.5, 1, 2 μM. E-F. Immunofluorescent staining of MCF7 (E) or HT1080 (F) cells with the antibodies to the indicated proteins and DAPI for DNA. Treatments: in E – CBL0137, 3μM, ARTIK-52, 1 μM – positive control for the replication-dependent DNA damage for 3 or 7 hours, F – CBL0137, 1 μM , CBL0100, 0.2 μM, bleomycin, 500 μg/ml for 3 or 7 hours. G. Quantitation of data in F. *** - t-test $p < 0.001$ from untreated control. Other comparisons are non-significant.

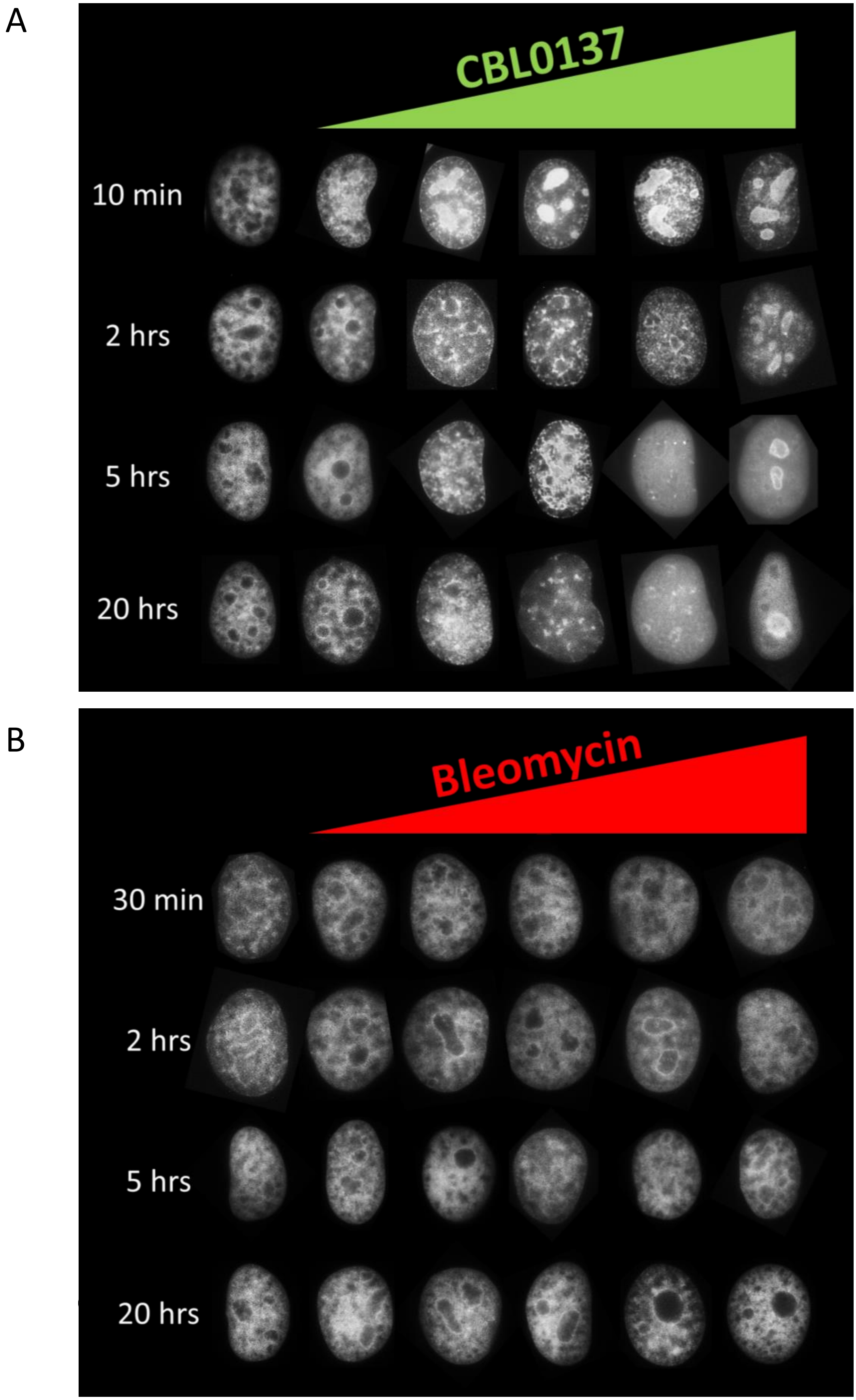

Supplementary Figure S2. Live-cell imaging of nuclei of HT1080 cells expressing histone H1 tagged with mCherry before and after treatment with CBL0137 (A) or bleomycin (B) at different time points after start of treatment. *Continued on the next page.*

Supplementary Figure S2. Live-cell imaging of nuclei of HT1080 cells expressing histone H1 tagged with mCherry before and after treatment with CBL0137 (A) or bleomycin (B) at different time points after start of treatment. Cells were plated in cell chambers with glass bottom. Next day they were treated with (A) CBL0137 (0, 0.3, 0.63, 1.25, 2.5, 5 μM) or (B) bleomycin (0, 63, 125, 250, 500, 1000 μg/ml). Brightness and size of individual nuclei were adjusted to compensate for their heterogeneity unrelated to the treatment. Images represent appearance of 70-100 % of interphase cells in population.

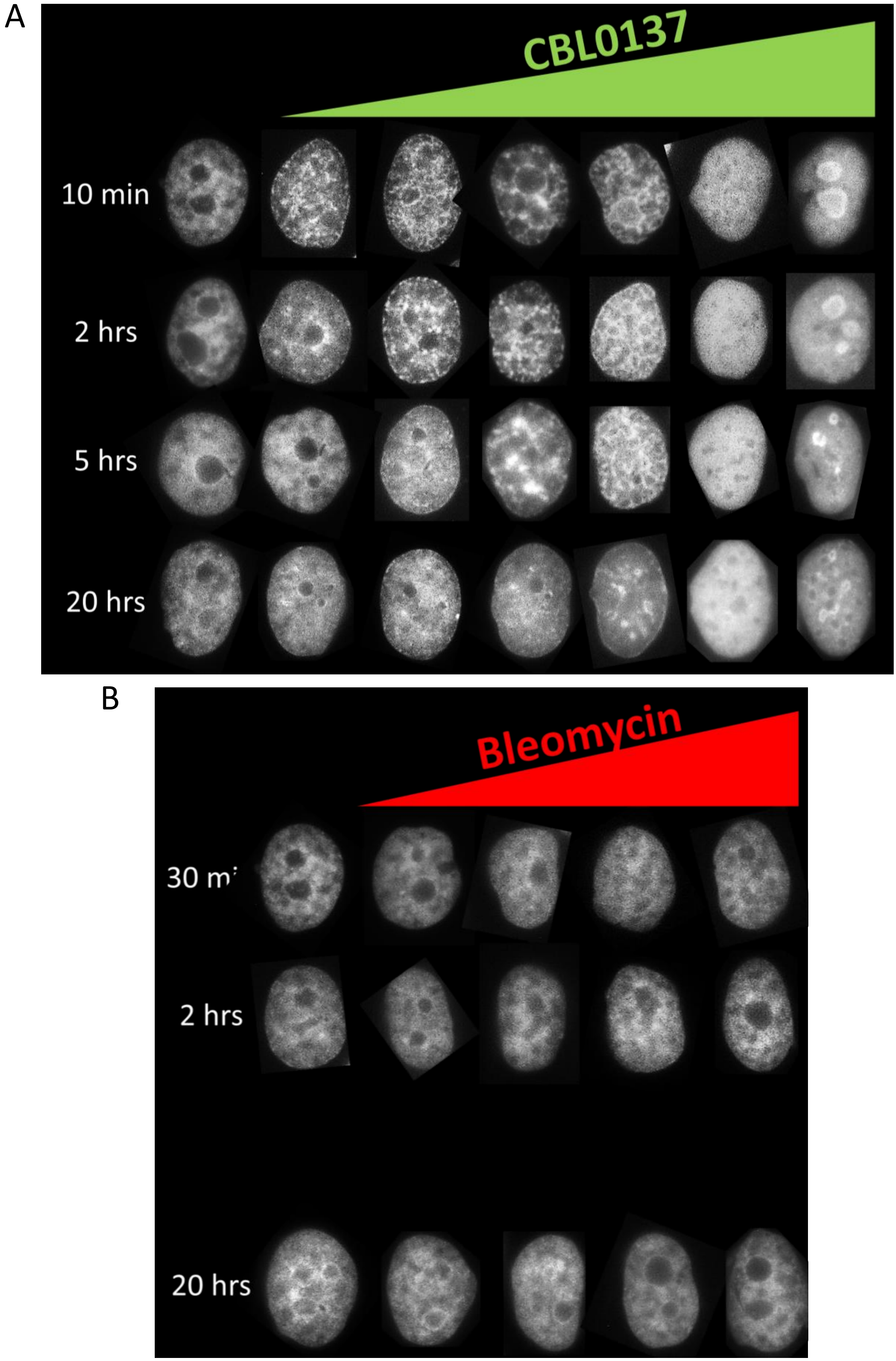

Supplementary Figure S3. Live-cell imaging of nuclei of HT1080 cells expressing histone H2B tagged with mCherry before and after treatment with CBL0137 (A) or bleomycin (B) at different time points after start of treatment. *Continued on the next page.*

Supplementary Figure S3. Live-cell imaging of nuclei of HT1080 cells expressing histone H2B tagged with mCherry before and after treatment with CBL0137 (A) or bleomycin (B) at different time points after start of treatment. Cells were plated in cell chambers with glass bottom. Next day they were treated with (A) CBL0137 (0, 0.3, 0.63, 1.25, 2.5, 5, 10 μM) or (B) bleomycin (0, 125, 250, 500, 1000 μg/ml). Brightness and size of individual nuclei were adjusted to compensate for their heterogeneity unrelated to the treatment. Images represent appearance of 70-100 % of interphase cells in population.

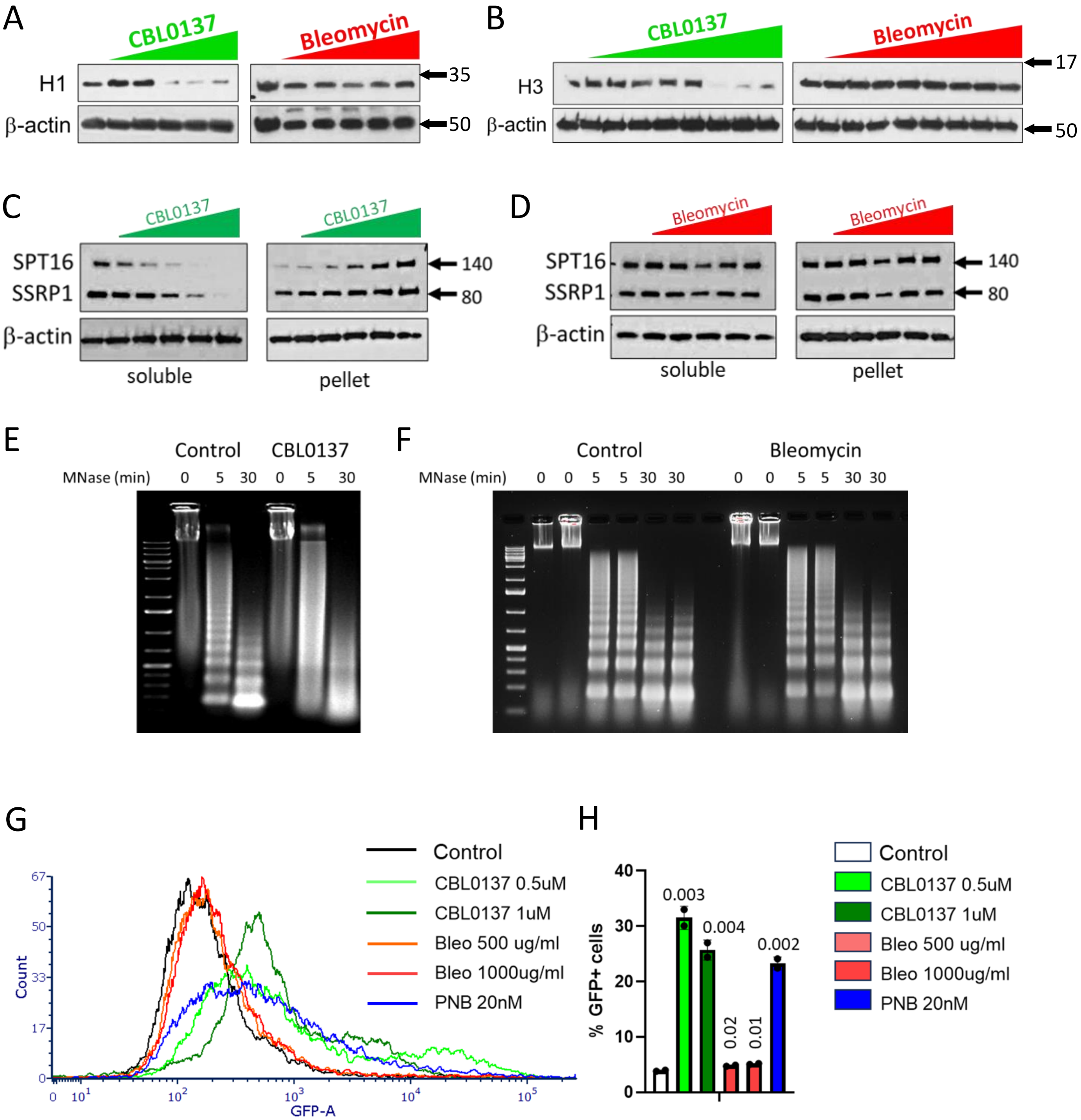

Supplementary Figure S4. Comparison of chromatin damage caused by different molecules. A-B. Histone eviction from chromatin. Western blotting of chromatin fractions of HT1080 cells. A. Cells were treated with CBL0137 (0. 0.63, 1.25, 2.5, 5, 10 μM) or bleomycin (0, 60, 125, 250, 500, 1000 μg/ml) and probed with antibodies to histone H1. B. Cells were treated with CBL0137 (0, 0.25. 0.5, 0.75, 1, 1.5, 2.5, 5, 10 μM) or bleomycin (0, 60, 125, 250, 500, 750, 1000, 1500, 2000 μg/ml) and probed with antibodies to histone H3. C-D. C-trapping of FACT. Western blotting of soluble and pelleted protein fractions from HT1080 cells treated with (C) CL0137 (0, 0.325, 0.63, 1.25, 2.5, 5 μM) or (D) bleomycin (0, 63, 125, 250, 500, 1000 μg/ml) for 24 hrs. The soluble fraction contains the nucleoplasm, and the pelleted fraction represents chromatin. *Continued on the next page.*

Supplementary Figure S3. Comparison of chromatin damage caused by different molecules. E-F. MNase digestion assay. Agarose gel electrophoresis of DNA isolated from nuclei of HT1080 cells treated with (E) 0.5 and 1 μM of CBL0137 or (F) 500 and 1000 μg/ml of bleomycin (in duplicates). Isolated nuclei were incubated with MNase for the indicated amount of time. Nuclei from untreated cells are shown as control. G-H. Desilencing of CMV promoter controlling expression of GFP in HeLa-TI cells. Cells were treated with the indicated drugs for 24 hrs, PNB – panobinostat, HDAC inhibitor used as positive control for chromatin desilencing. G. Histogram of GFP fluorescence obtained using flow-cytometry. One replicate is shown. H. Quantitation of GFP fluorescence from the same experiment as in G. Bars – mean of two replicates. Dots – individual values. Number above bars are p-values between treatment and control, t-test.

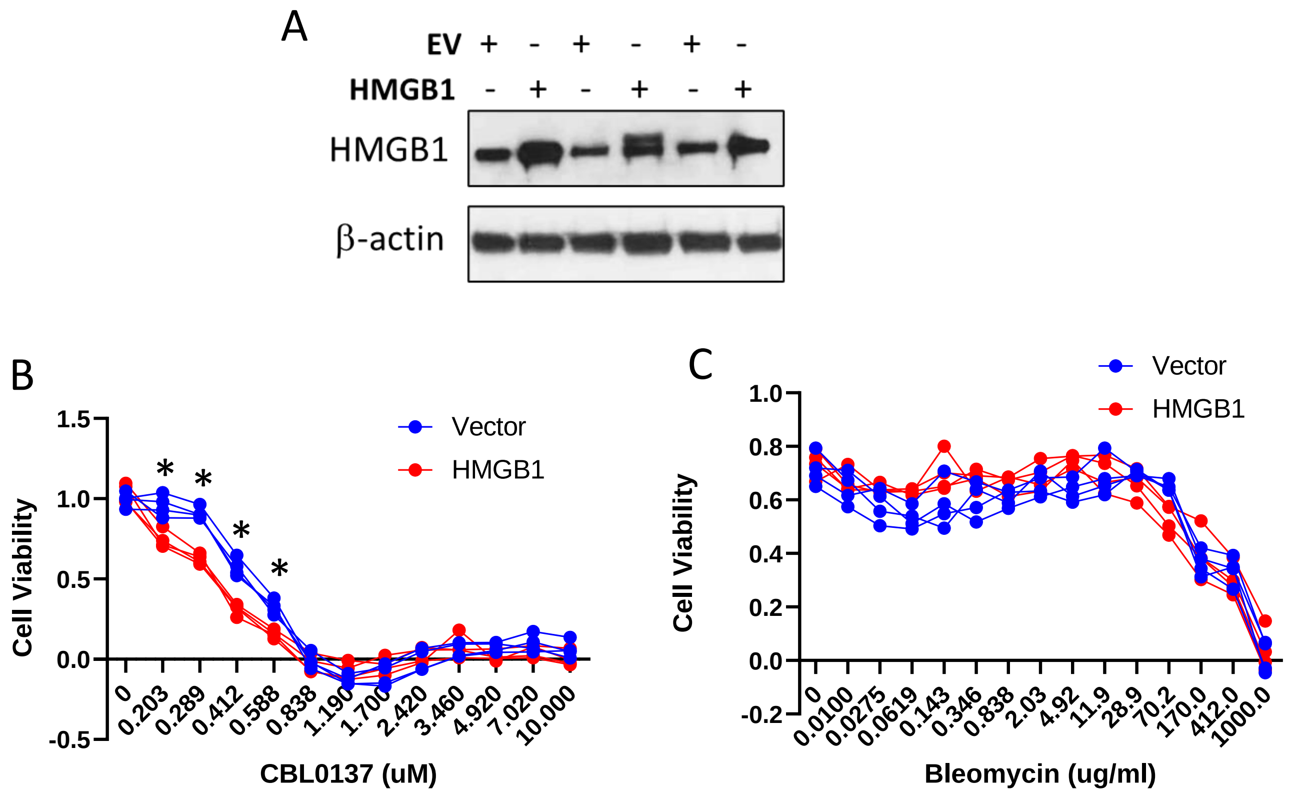

Supplementary Figure S5. CBL0137 but not bleomycin are more toxic to cells overexpressing HMGB1. A. Western blotting of protein extracts of HT1080 cells transduced with empty vector of HMGB1 expressing construct and selected with puromycin. Three replicates of each transduction are shown. B, C. Viability of HT1080 cells transduced with empty vector or HMGB1 expressing construct and treated with different concentrations of CBL0137 or bleomycin for 72 hours. Curves are individual replicates within experiment. n = 4. * p-value < 0.05, t-test.

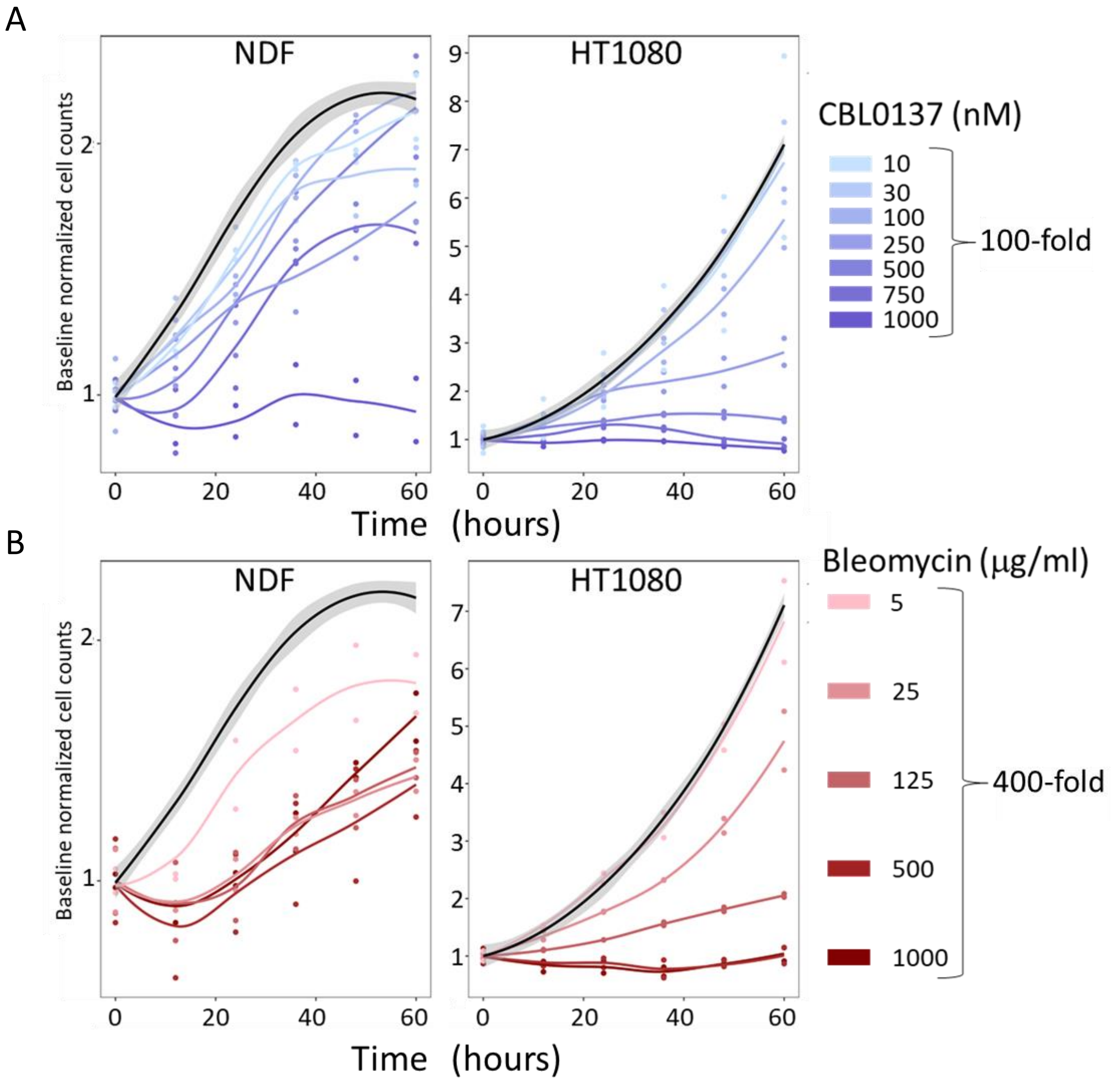

Supplementary Figure S6. Time-dependent effects of CBL0137 and bleomycin on the growth of NDFs and HT1080 cells. Cells were plated in 384-well plates in triplicate. The next day, the cells were treated with different doses of CBL0137 (A) or bleomycin (B). Cell number was evaluated by automatically counting of cells every 12 hr by a Cytation 5 Cell Imaging Multimode Reader using Gen5 ImagePrime software. Three replicates per condition are shown as dots. Untreated control cells are shown as a black line with a grey area representing the standard error for ten replicates. Cell counts were normalized to a time point immediately after drug addition (approximately 5 min).

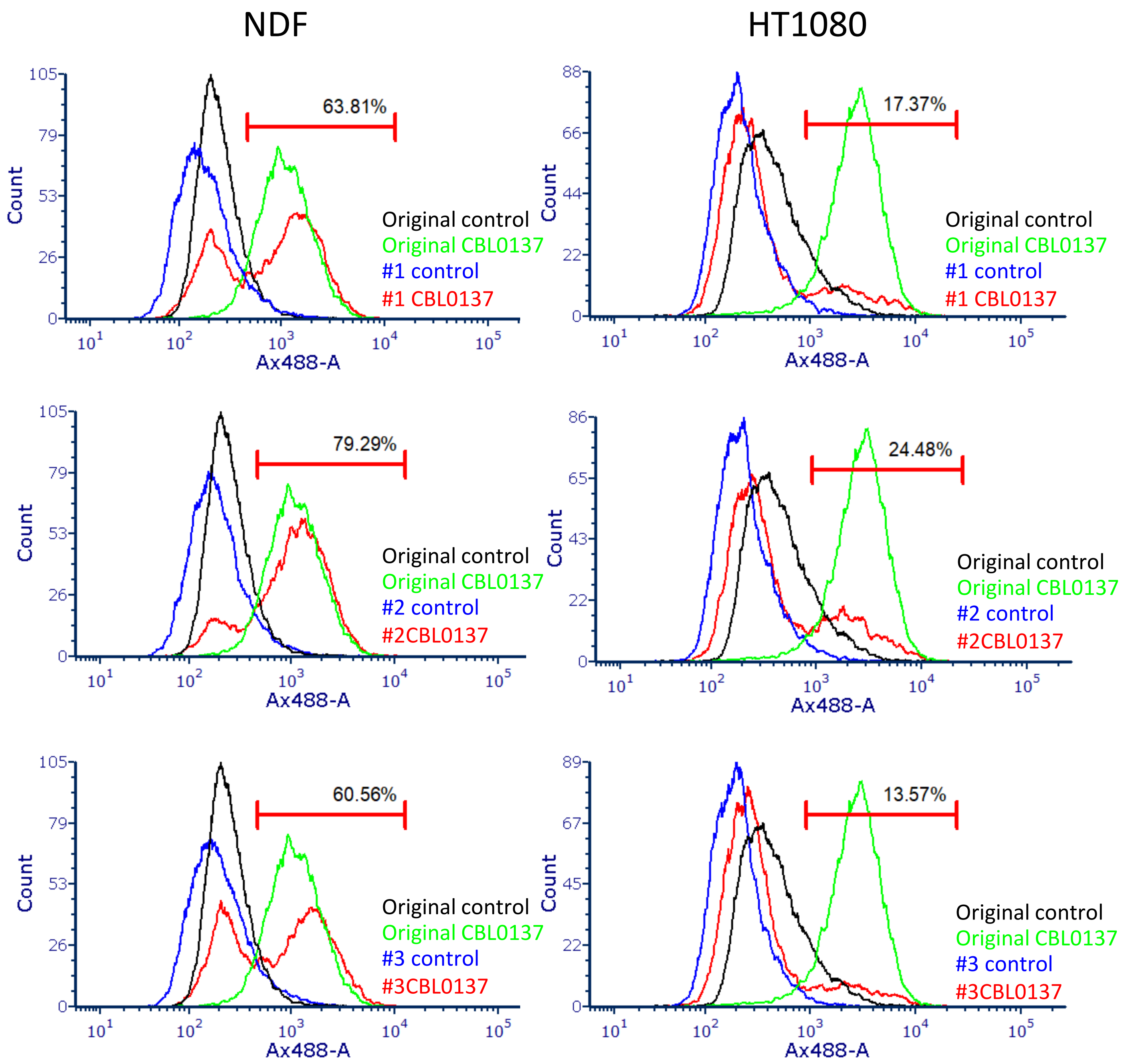

Supplementary Figure S7. Assessment of the proportion of p53-negative cells following electroporation with Cas9 and gRNAs to *TP53* gene. NDFs and HT1080 cells, parental and post-electroporation, were treated with 1 μM CBL0137 for 24 hr to induce p53. Treated and untreated cells were stained for p53 and analyzed by flow cytometry. Three slightly different electroporation regimens were used (#1, #2, and #3). The marker shows the position of the p53-positive cells in the parental cells after CBL0137 treatment. The percentage above each marker indicates the proportion of p53-positive cells after electroporation.

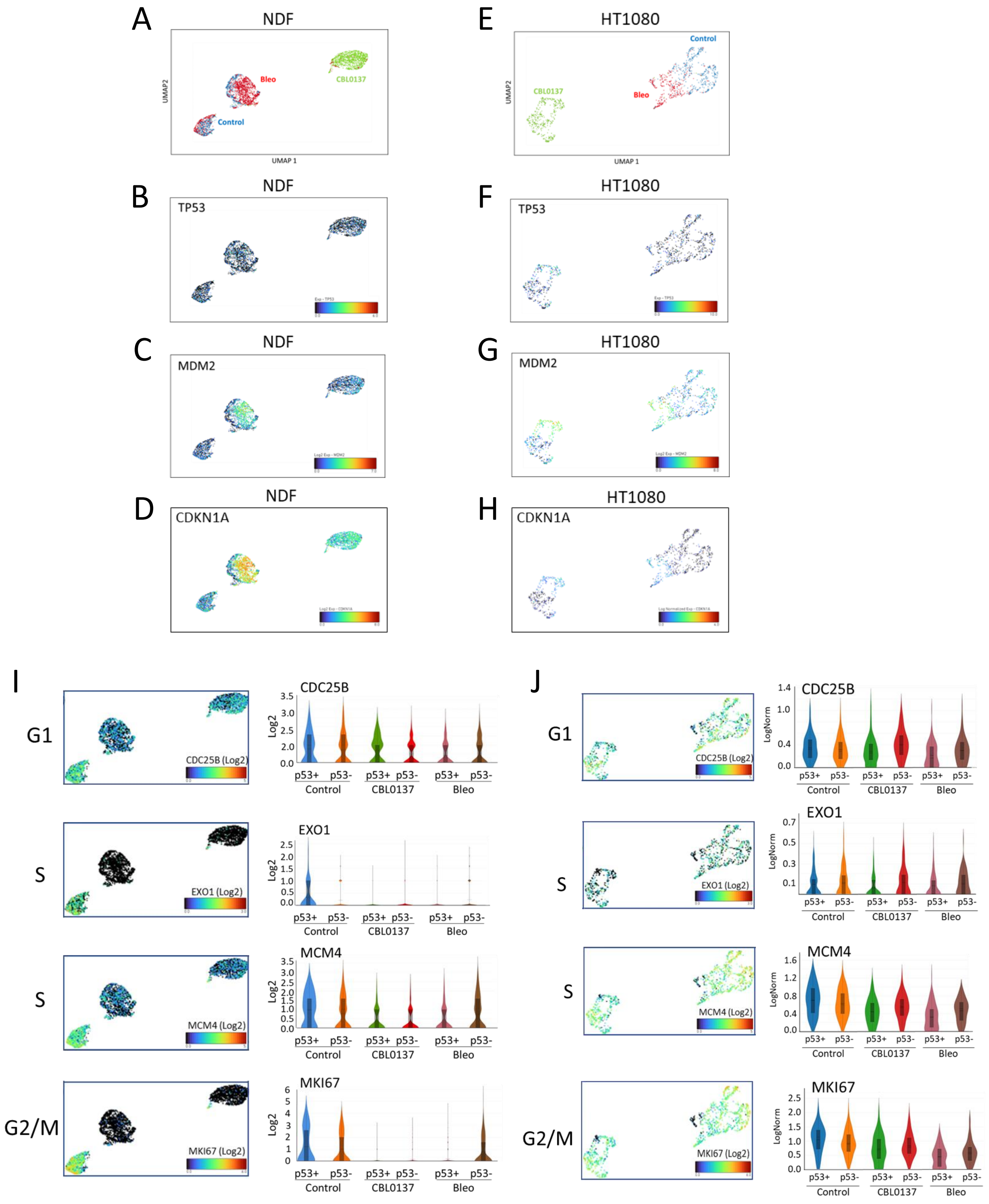

Supplementary Figure S8. Analyses of scRNA-seq data from NDFs (A-D, I) and HT1080 (E-H, J) cells. A-H. UMAP plots showing position of cells depending on treatment (A, E), or expression of the following genes, *TP53* (B, F), *MDM2* (C, G) and *CDKN1A* (D, H). I, J. UMAP and violin plots showing the expression of markers for the G1, S, and G2/M phases of the cell cycle in NDF (I) and NT1080 (J) cells under different conditions.

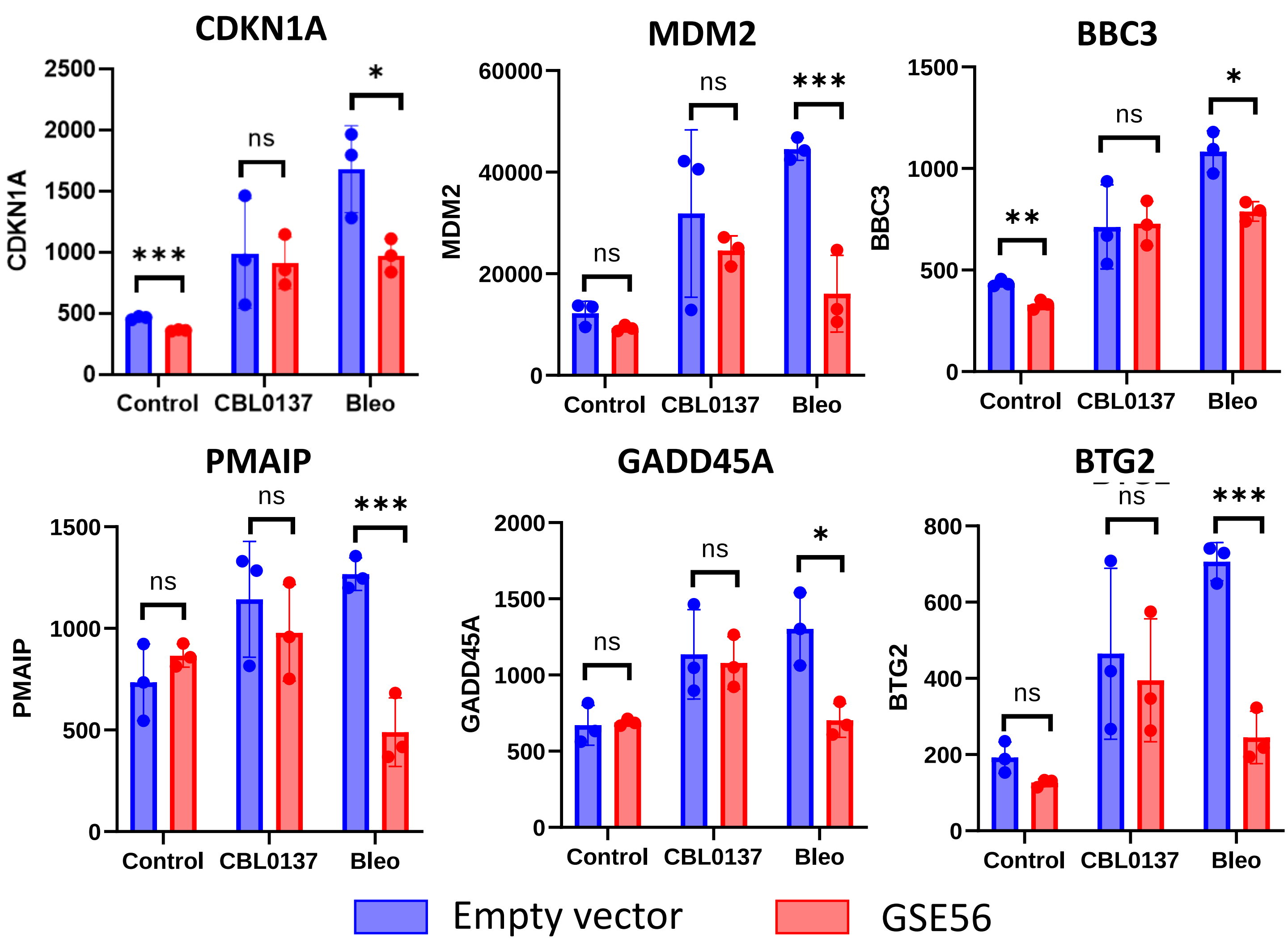

Supplementary Figure S9. Transcription of p53 target genes in response to DD and CD. Normalized data for nascent RNA-seq from HT1080 cells transduced with empty vector or p53 dominant-negative mutant GSE56 treated with 0.6 µM CBL0137 or 400 µg/ml bleomycin for 24 hr. Controls were left untreated. Data are presented as the mean ± SD (n = 3 replicates). *p < 0.05, **p < 0.01, ***p < 0.005, ns, not significant by the paired Student's t-test, functional p53 vs. inactive p53.

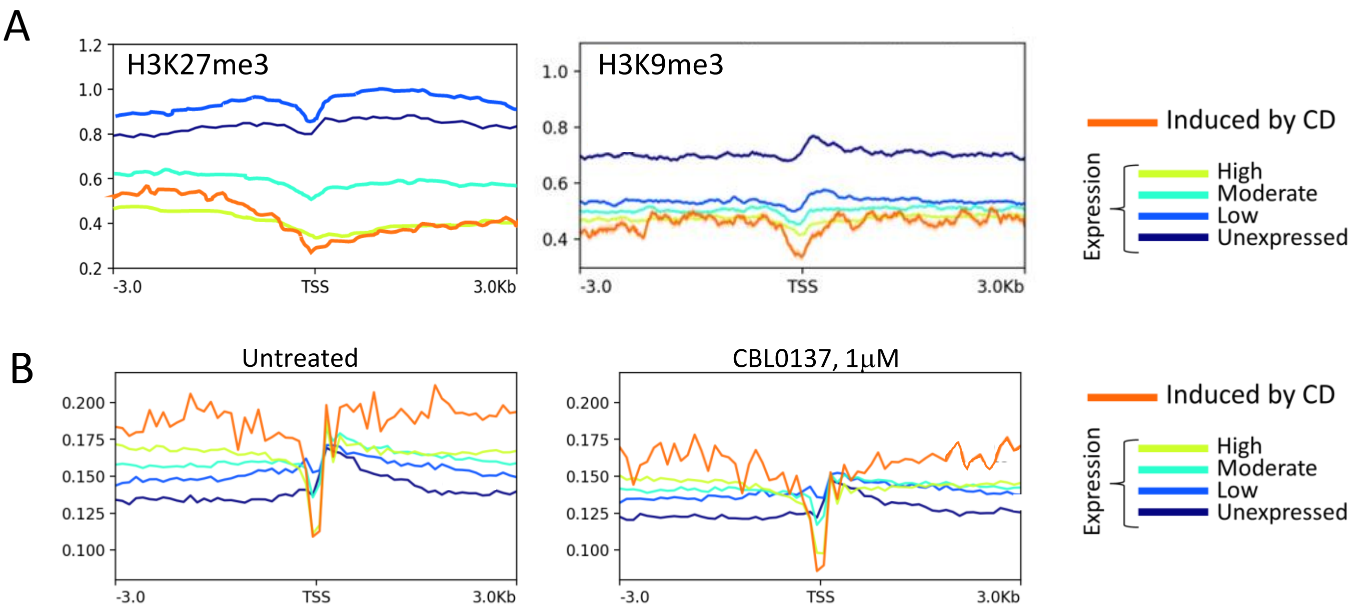

Supplementary Figure S10. Comparison of chromatin state around TSS of genes induced by CD and genes transcribed at different levels. A. Metagene profiles of repressive histone post-translational modifications for different categories of genes in untreated NDF cells . B. Metagene profiles of DNA protected from Mnase digestion in untreated HT1080 cells and HT1080 cells treated with CBL0137 for 1 hour.

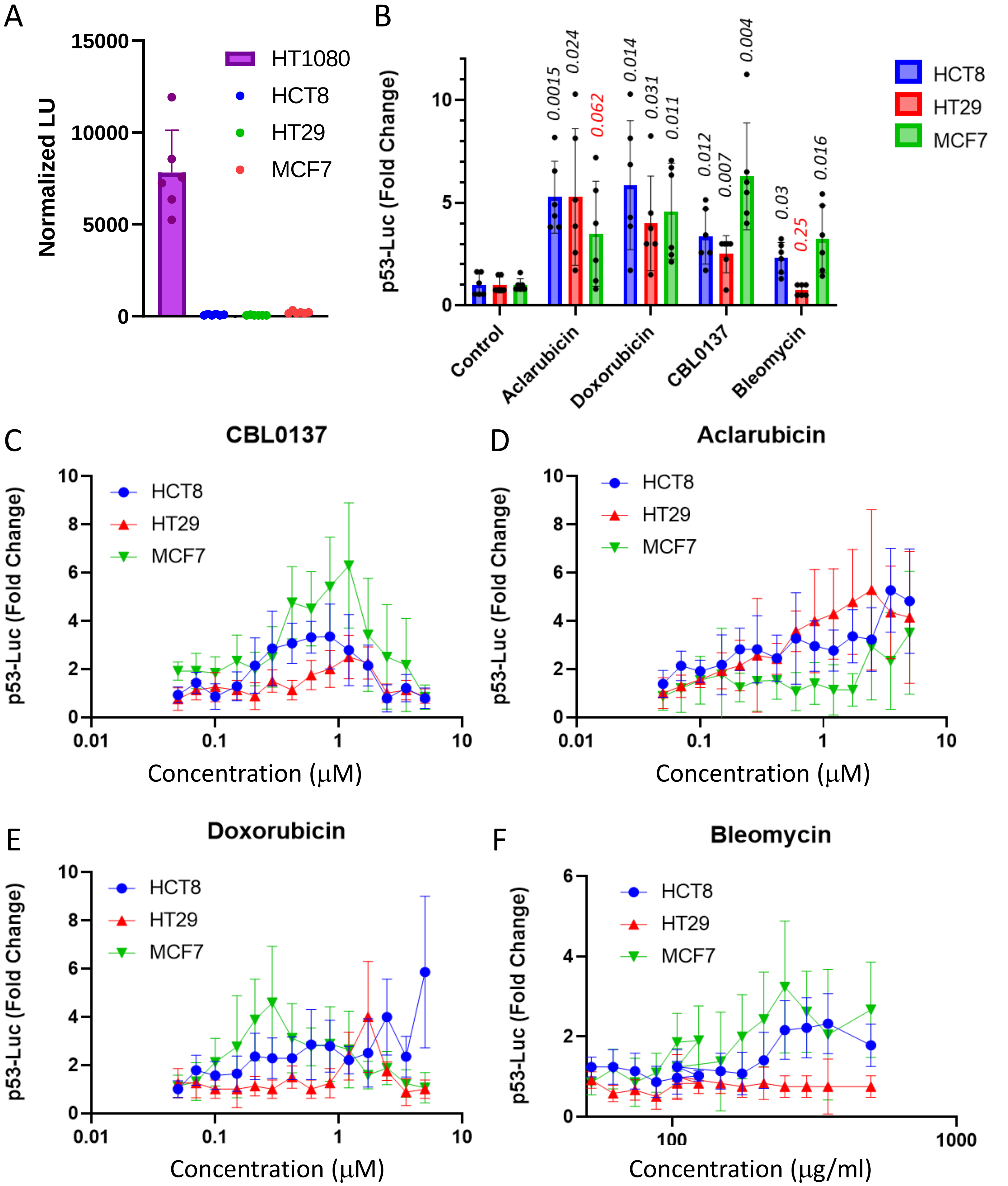

Supplementary Figure S11. Activation of p53 reporter by ChrD and DnaD compounds in p53 wild type (HT1080, HCT8, MCF7) and p53 mutant (HT29). A-D. Basal activity of the reporter in untreated cells normalized by cell number. B. Maximal induction of the reporter. For each compound concertation causing maximal mean activation of p53 reporter is shown together with individual values from 6 replicates. Numbers are p-values of Student t-test between treated and untreated (control) samples. C-F. Dose dependent response curves for ChrD only compounds CBL0137 (C), aclarubicin (D), ChrD and DnaD compound doxorubicin (E), and DD only compound bleomycin (F). Mean +/- SE, n=6.
